# Supplementary material for: The rapid evolution of lungfish durophagy
Source: Nat Commun. 2022 May 2;13:2390. doi: 10.1038/s41467-022-30091-3 (PMC9061808; doi:10.1038/s41467-022-30091-3)
Supplement: Supplementary file 1 — Supplementary Information [file 41467_2022_30091_MOESM1_ESM.pdf]

SUPPLEMENTARY INFORMATION

Supplementary Notes

µCT analysis of specimens of *Youngolepis* revealed additional information on structures described in earlier reports[1,2]. Brief redescriptions of these structures are given here as complements to previous accounts, with an emphasis on new features apparent in our scans.

Vomer and parasphenoid

V28376 preserves the left vomer (Supplementary Figure. 1a), which lies anterior to the other dermal ossifications of the palate. Its dorsal face is flat and has a rounded, triangular outline. The ventral (buccal) surface bears a robust fang flanked by two smaller teeth and associated with a replacement pit (Supplementary Figure. 1a). In V28375 (Fig. 2a), the parasphenoid is firmly attached to the ethmosphenoid, whereas the parasphenoid in V28376 (Supplementary Figure. 1a) is isolated. Both examples correspond to the third parasphenoid morphotype identified by Chang[1]: a rectangular outline with slightly concave lateral margins and a convergent posterior margin. The ventral (buccal) face of the parasphenoid is covered by denticles. The denticles from the anterior part and lateral margins are larger than those situated in the posterior part of the bone. An oval buccophypophysial canal pierces the parasphenoid (Supplementary Figure. 1a).

Lower jaw

The two specimens both preserve the left and right mandibles in their natural positions (Supplementary Figure. 1c–h). The lower jaws agree well with isolated specimens attributed to *Youngolepis* sp.[2]. Dorsally, the lower jaw has three coronoids (Supplementary Figure. 1c, g). Each bears a blunt fang, flanked laterally by many randomly arranged small teeth. The dorsal margin of the dentary bears a row of regularly spaced, larger teeth that extends from the adductor fossa to the anterior end of the lower jaw (Supplementary Figure. 1c, g). This differs from conditions in *Achoania*, *Psarolepis,* and *Youngolepis* sp.[2], where the symphysis bears no dentary teeth. The prearticular is thin and elongate (Supplementary Figure. 1e, g). The dorsal margin of the prearticular bears rows of small teeth (Supplementary Figure. 1h), with those adjacent to the coronoid fangs being the largest. The oval adductor fossa constitutes about 40 percent of total mandibular length (Supplementary Figure. 1c, g). The biconcave glenoid fossa (Supplementary Figure. 1c, g) lies behind the adductor fossa, and marks the articulation with the quadrate.

Much of the lower jaw is of the same width, being only slightly narrower at the distal and proximal ends (Supplementary Figure. 1d, h). On the external surface, no sutures are apparent between the dentary and infradentaries, or between the infradentaries themselves. Three infradentary foramina pierce the lateral face of the jaw (Supplementary Figure.1d), and presumably indicate the boundaries between the infradentaries as in many porolepiforms[3]. A row of small pores along the lower margin of the lateral surface of the mandible marks the course of the mandibular canal[3-5]. The antero-medial lamina of the dentary is prominent and has a symphysial pit (Supplementary Figure. 1e). Although the longitudinal grooves and ridges on the antero-medial lamina of the dentary suggest that *Youngolepis* may have the parasymphysial dental plate or whorl as in porolepiforms, no such bone is apparent in the articulated specimens. A groove extends along the ventral margin of the mandible between the prearticular and infradentaries, forming a trough for accommodating submandibular bones (Supplementary Figure. 1f). No evidence of numerous, small buccal toothplates can be found as in porolepiforms and *Powichthys*[3,6].

Supplementary Figures

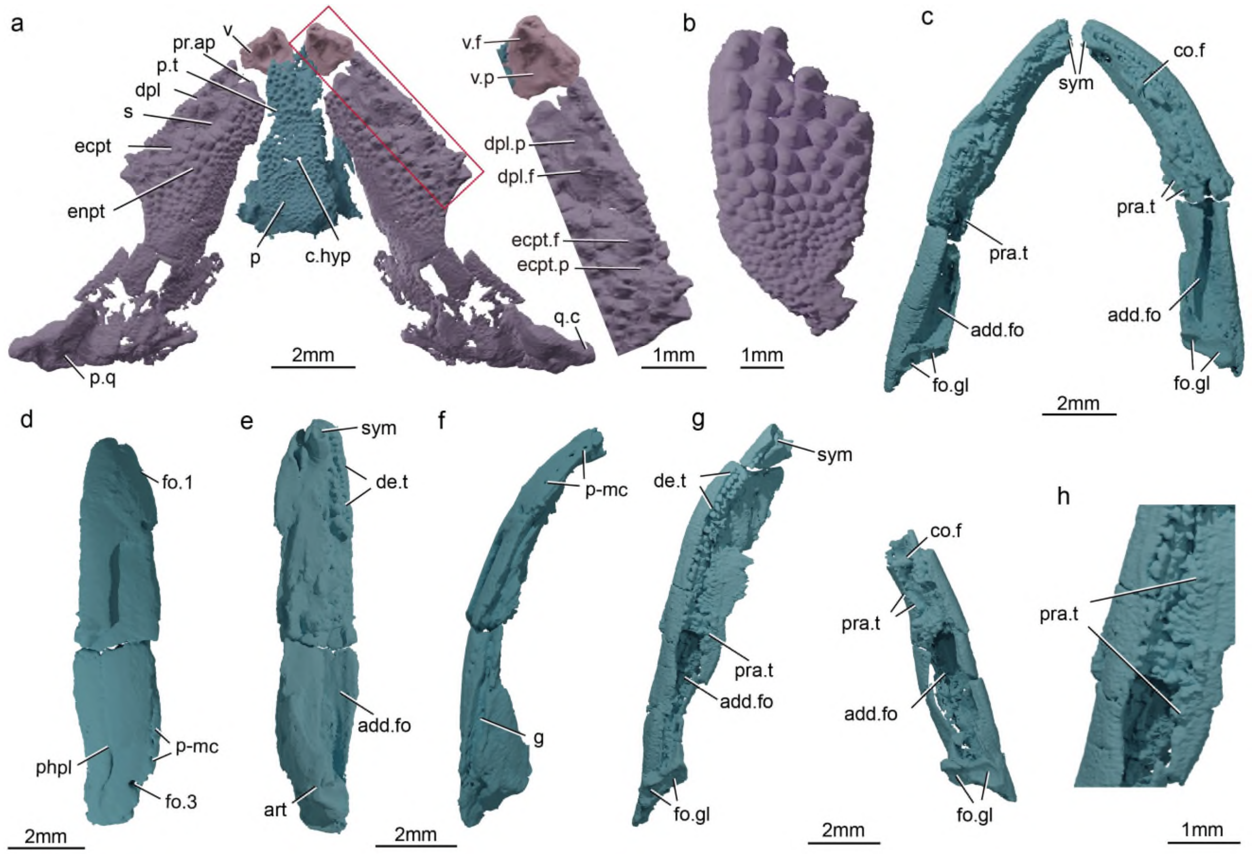

Supplementary Fig. 1 *Youngolepis praecursor*, specimens IVPP V28375 and V28376 and *Diabolepis speratus*, specimen V28420.2. **a** Virtual rendering of palatoquadrate complex, vomer and parasphenoid of V28376 in ventral view. Palatoquadrate mirrored. Image to the right shows enlargement of area shown in red box, highlighting ectopterygoid and palatine fangs and their associated replacement pits. **b** Virtual rendering of left entopterygoid tooth plate of *Diabolepis*. **c–g** Virtual rendering of lower jaws of V28375 in dorsal (**c**), lateral (**d**), median (**e**), and ventral (**f**) view. **g** Virtual rendering of lower jaws of V28376 in dorsal view. **h** Enlarged view of prearticular dentition in **f**. add.fo, adductor fossa; art, articular; c.hyp, buccophypophysial canal opening; co.f, coronoid fangs; de.t, teeth on the dentary; dpl, dermopalatine; dpl.f, dermopalatine fang; dpl.p, dermopalatine fang replacement pit; ecpt, ectopterygoid; ecpt.f, ectopterygoid fang; ecpt.p, ectopterygoid fang replacement pit; enpt, entopterygoid; fo.gl, glenoid fossa; fo.1, first infradentary foramen; fo.3, third infradentary; g, groove; p, parasphenoid; p-mc, preoperculo-mandibular sensory canal; phpl, posterior horizontal pit-line; p.q, pars quadrata; p.t, parasphenoid teeth; pr.ap, autopalatine process; pra.t, prearticular teeth; q.c, quadrate condyle; s, suture; sym, symphysis; v, vomer; v.f, vomerine fang; v.p, vomerine fang replacement pit.

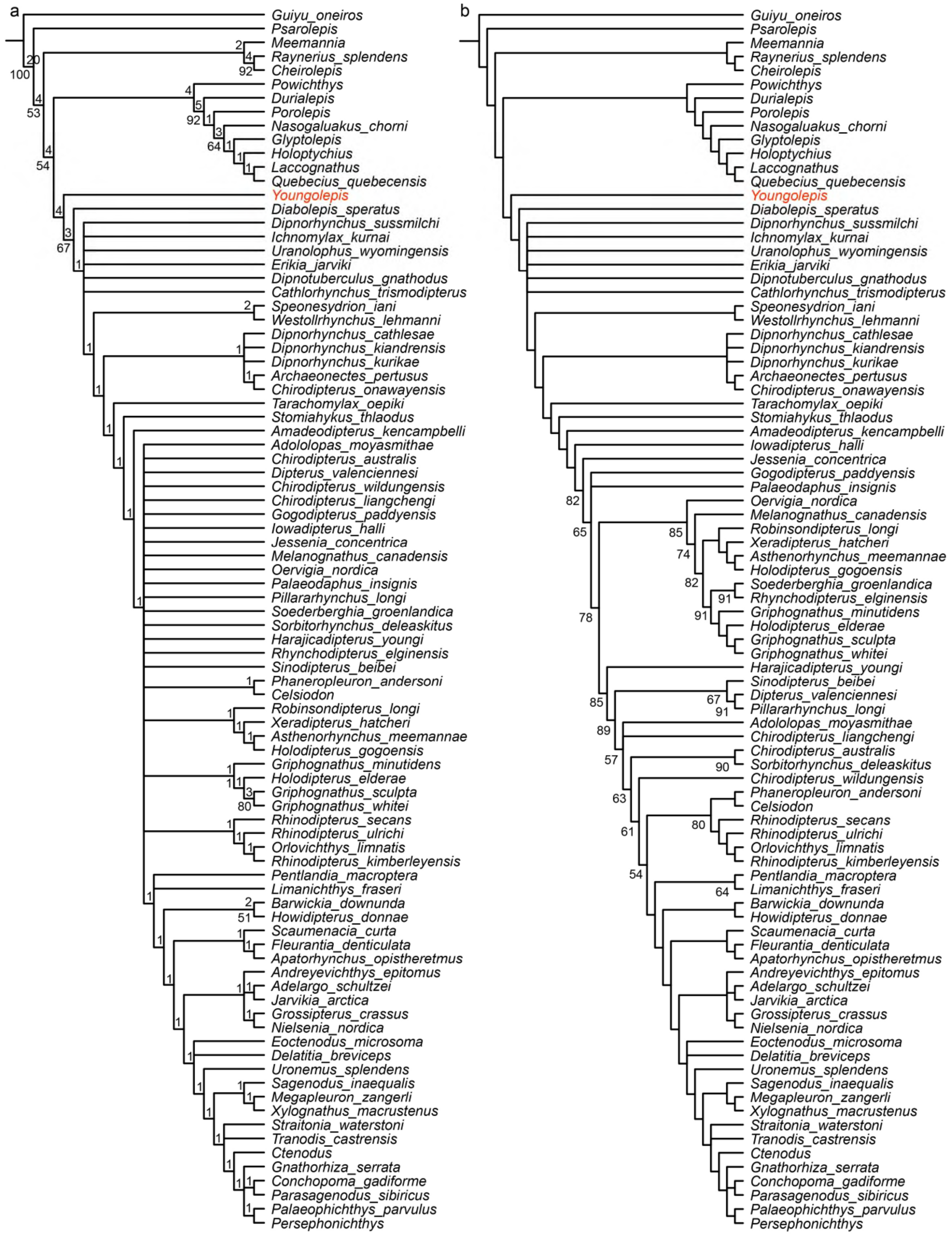

Supplementary Fig. 2 Phylogenetic relationships of dipnomorphs. (a) The strict consensus tree of 5824 most parsimonious trees of 1157 steps (CI=0.270; RI=0.629). Numbers above and below branches denote Bremer support and Bootstrap values (≥20% are shown). (b) 50% Majority-rule consensus tree. Numbers on branches indicate the percentage of most-parsimonious trees that contain a particular clade (100% unless otherwise indicated).

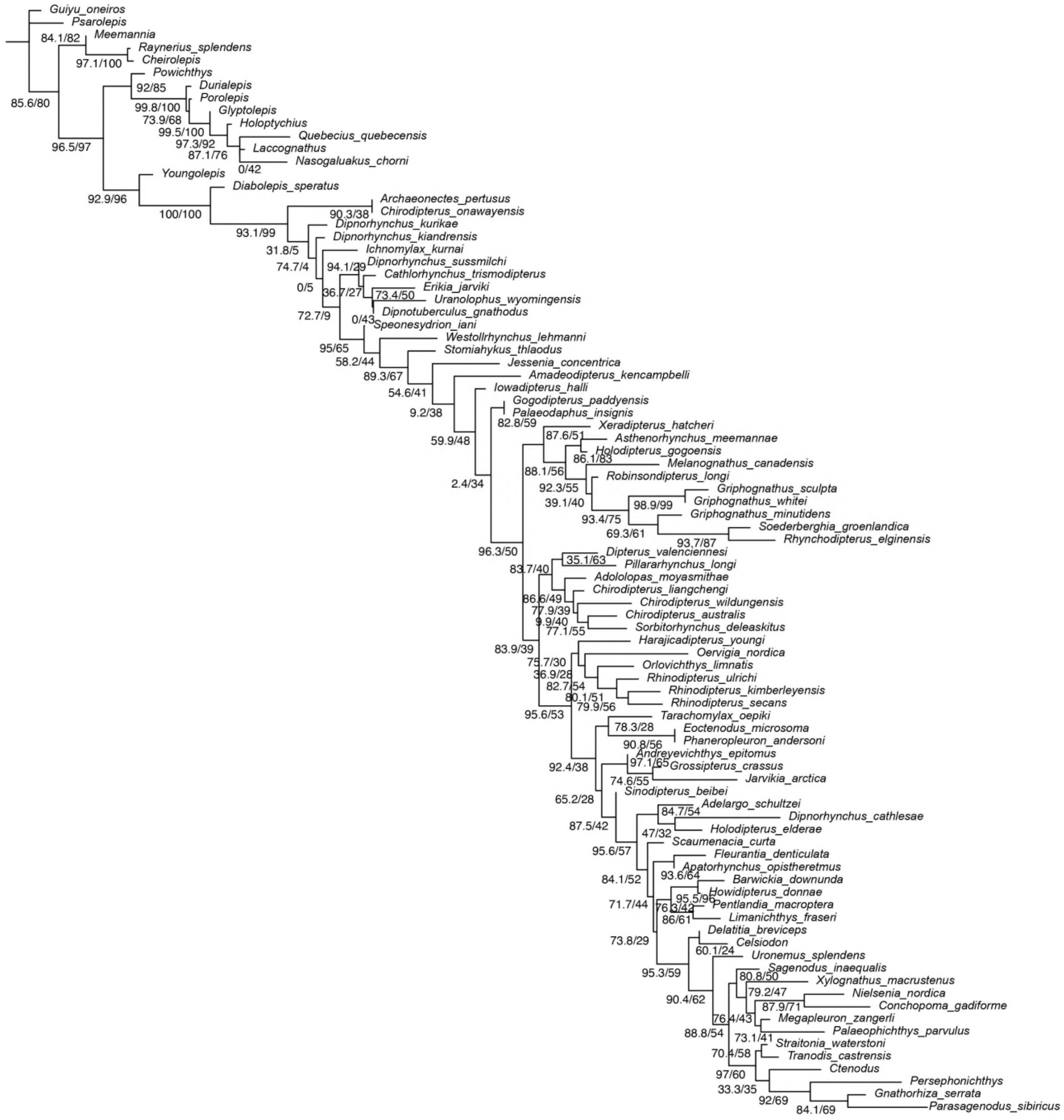

Supplementary Fig. 3 Maximum-likelihood tree. Number near the nodes are Shimodaira-Hasegawa-like approximate likelihood ratio test (SH-aLRT) and ultrafast bootstrap (UFB) from maximum likelihood analysis. SH-aLRT ≥ 85 or UFB ≥ 95 represent strong support.

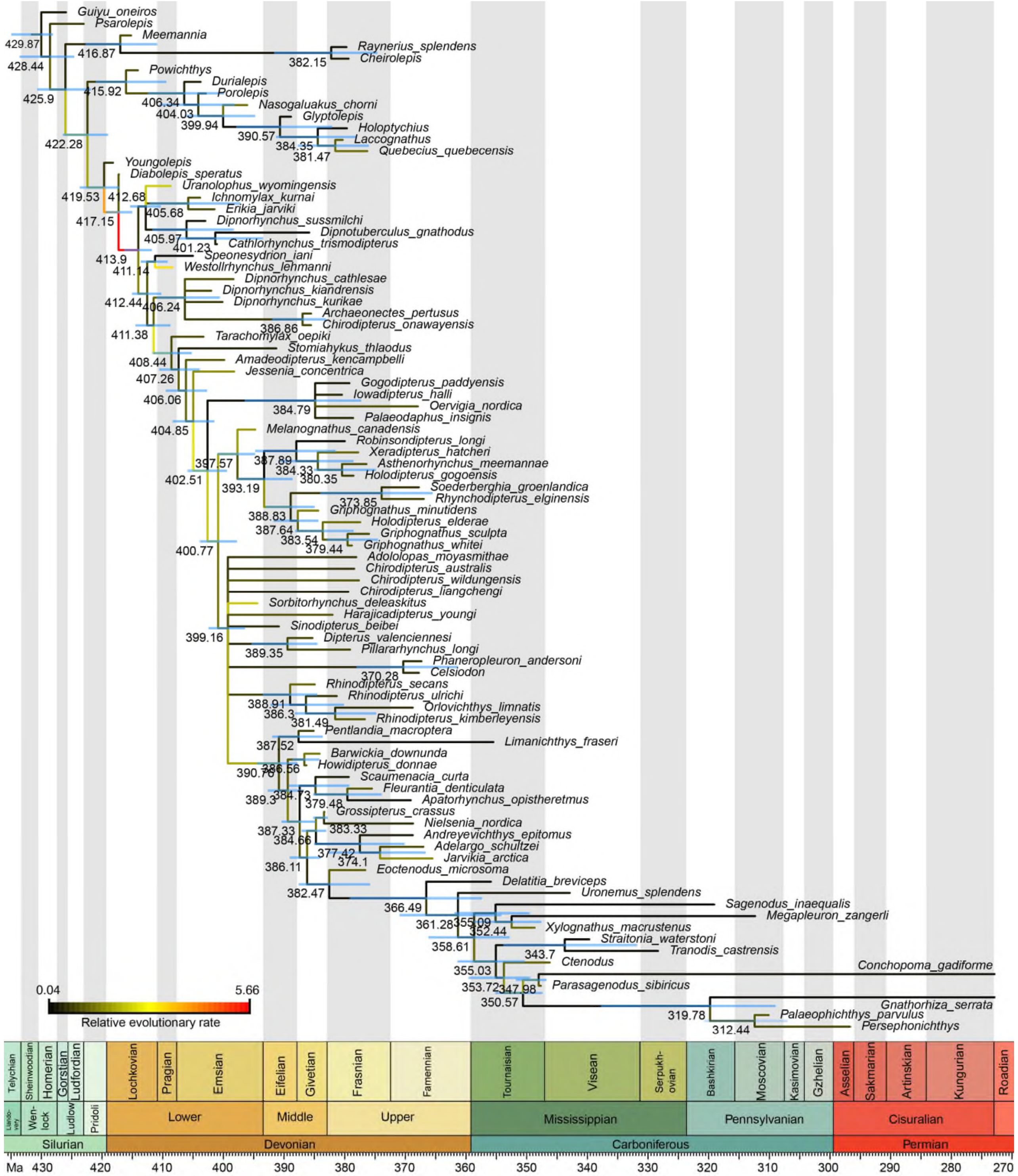

Supplementary Fig. 4 Time-calibrated phylogeny (constrained with strict consensus topology from parsimony analyses) obtained using IGR emphasizing relationships among Palaeozoic Dipnomorpha. The node ages are the posterior medians, with blue bars for each node representing 95% HPD intervals of age estimates. The color of the branch represents the mean relative clock rate along that branch.

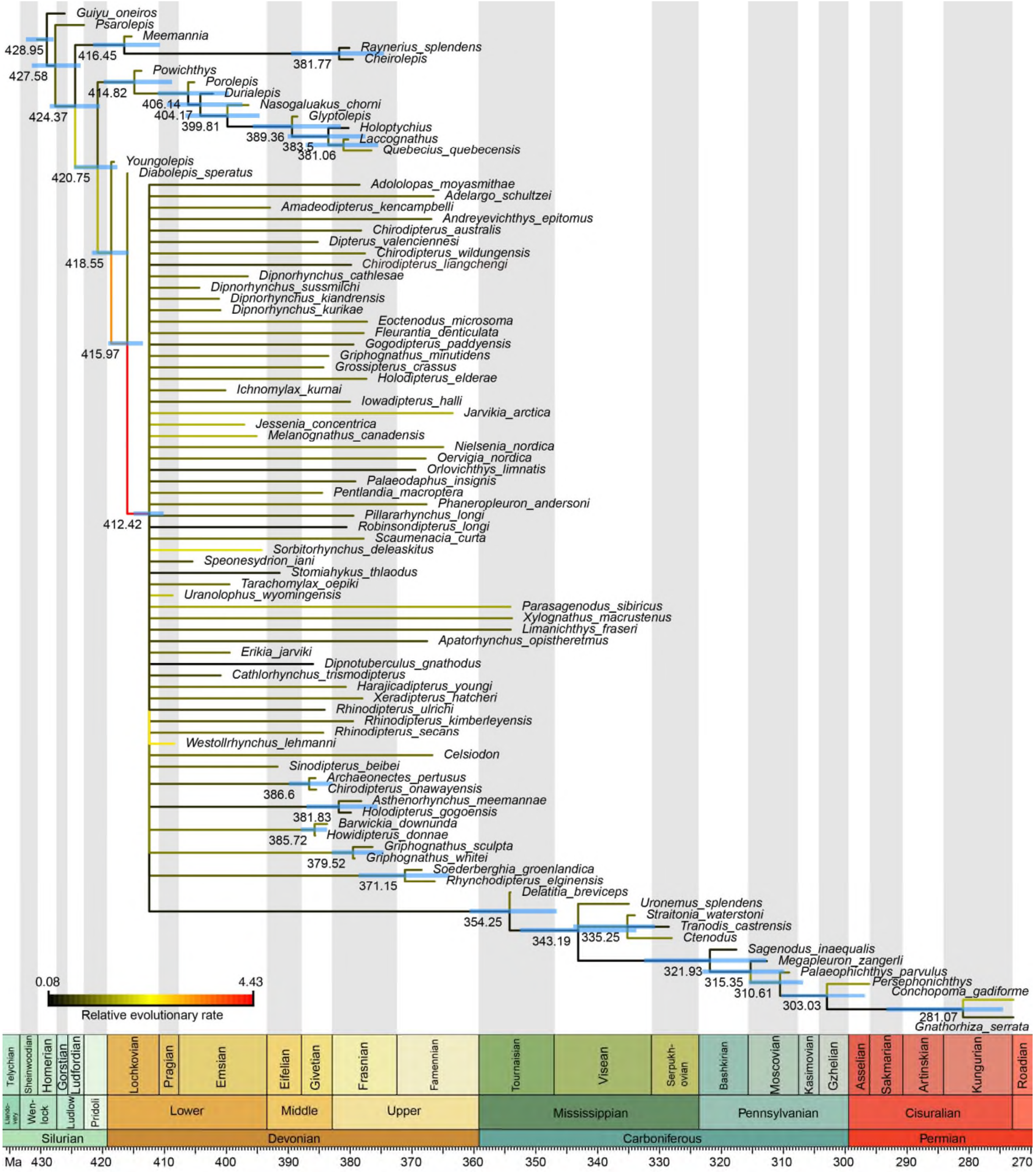

Supplementary Fig. 5 Time-calibrated phylogeny (no topological constraint) obtained using IGR emphasizing relationships among Palaeozoic Dipnomorpha. The node ages are the posterior medians, with blue bars for each node representing 95% HPD intervals of age estimates. The color of the branch represents the mean relative clock rate along that branch.

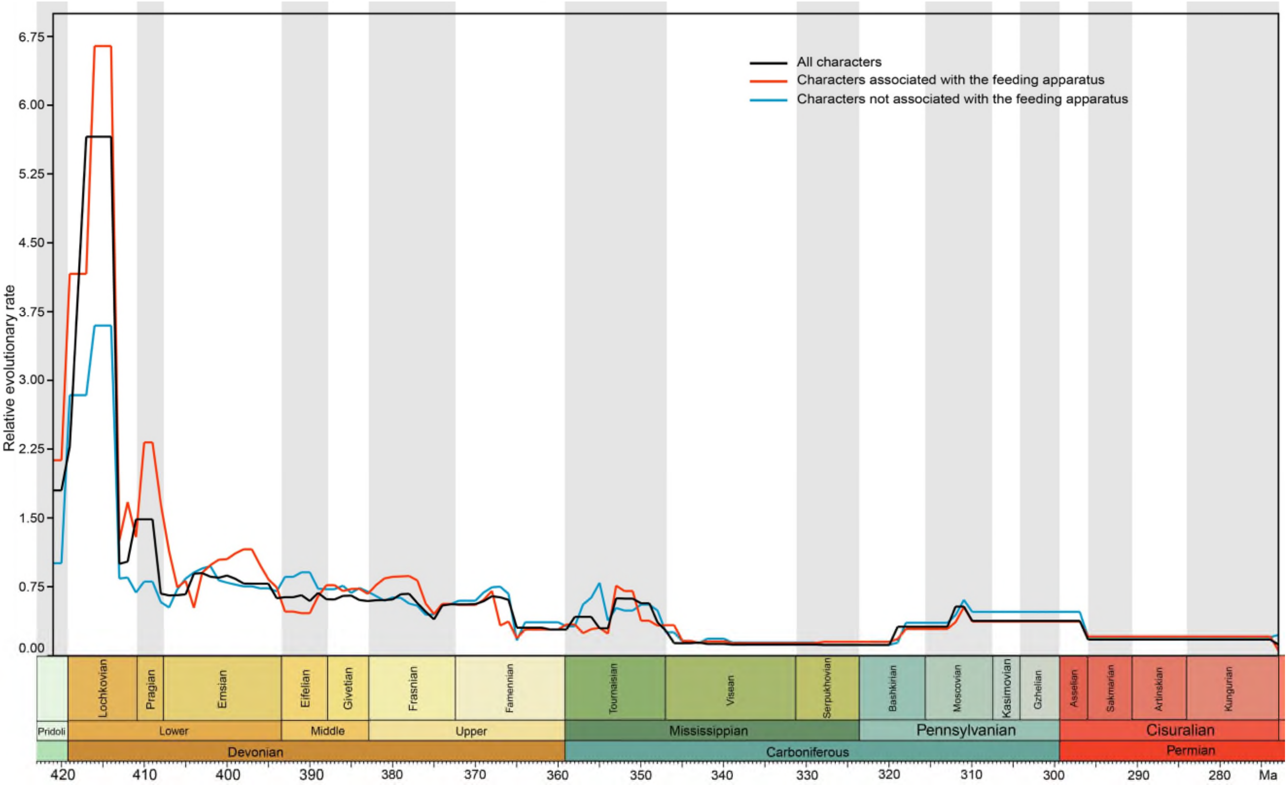

Supplementary Fig. 6 IGR relative rates of phenotypic evolution for all dipnomorphs more closely related to crown lungfishes than to Porolepiformes. Partitioned analyses were performed with a topological constraint matching the strict consensus parsimony solution (as in Supplementary Fig. 4).

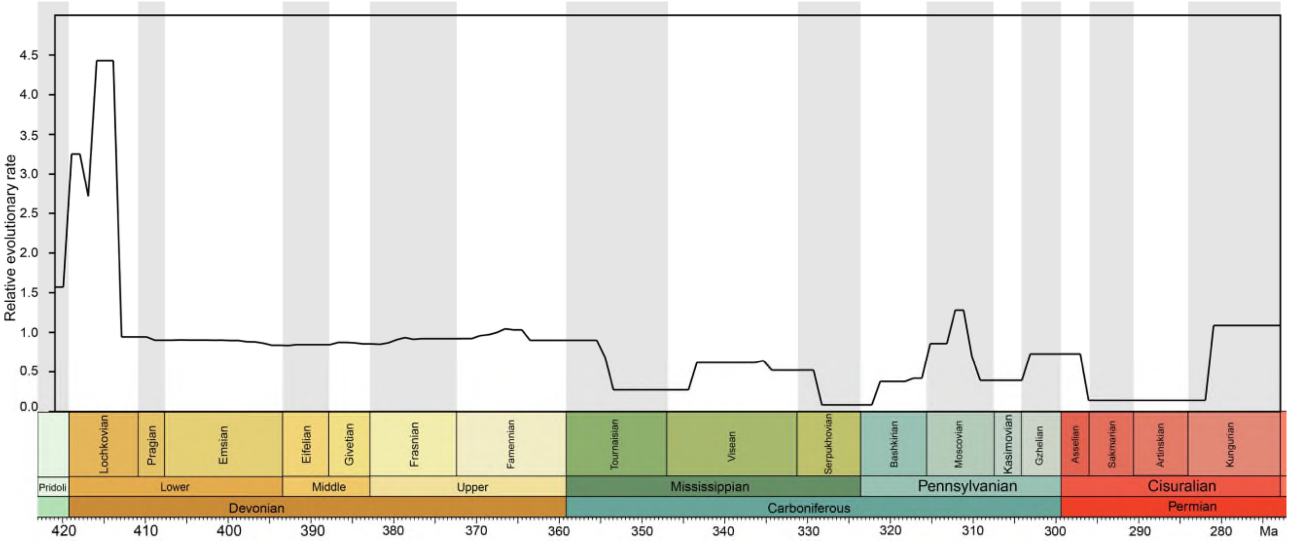

Supplementary Fig. 7 IGR relative rates of phenotypic evolution for all dipnomorphs more closely related to crown lungfishes than to Porolepiformes. Unpartitioned analyses with no constraint (as in Supplementary Fig. 5).

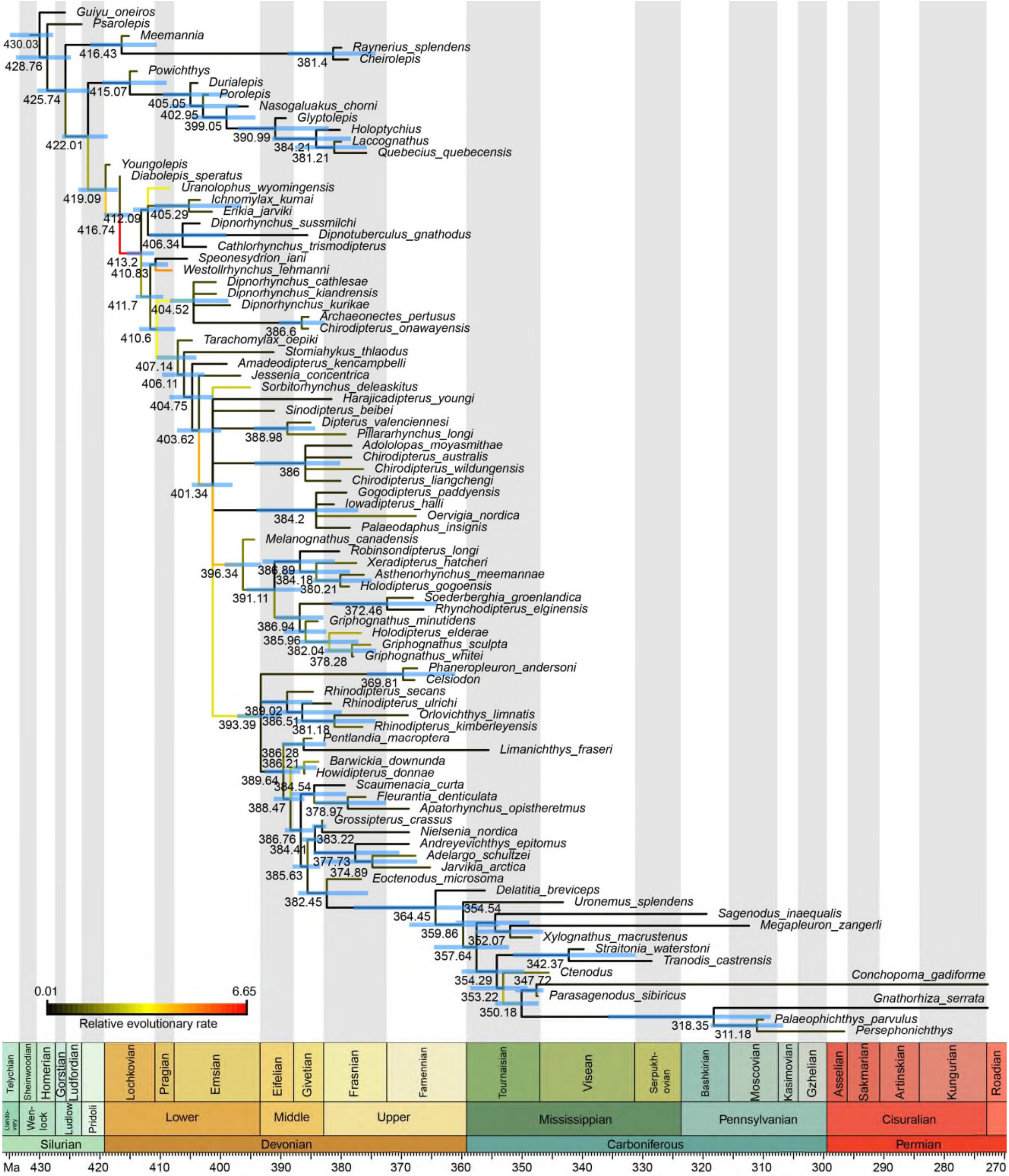

Supplementary Fig. 8 IGR evolutionary rate of characters associated with the feeding apparatus. The node ages are the posterior medians, with blue bars for each node representing 95% HPD intervals of age estimates. The color of the branch represents the mean relative clock rate at that branch. Partitioned analyses were performed with a topological constraint matching the strict consensus parsimony solution (as in Supplementary Fig. 4).

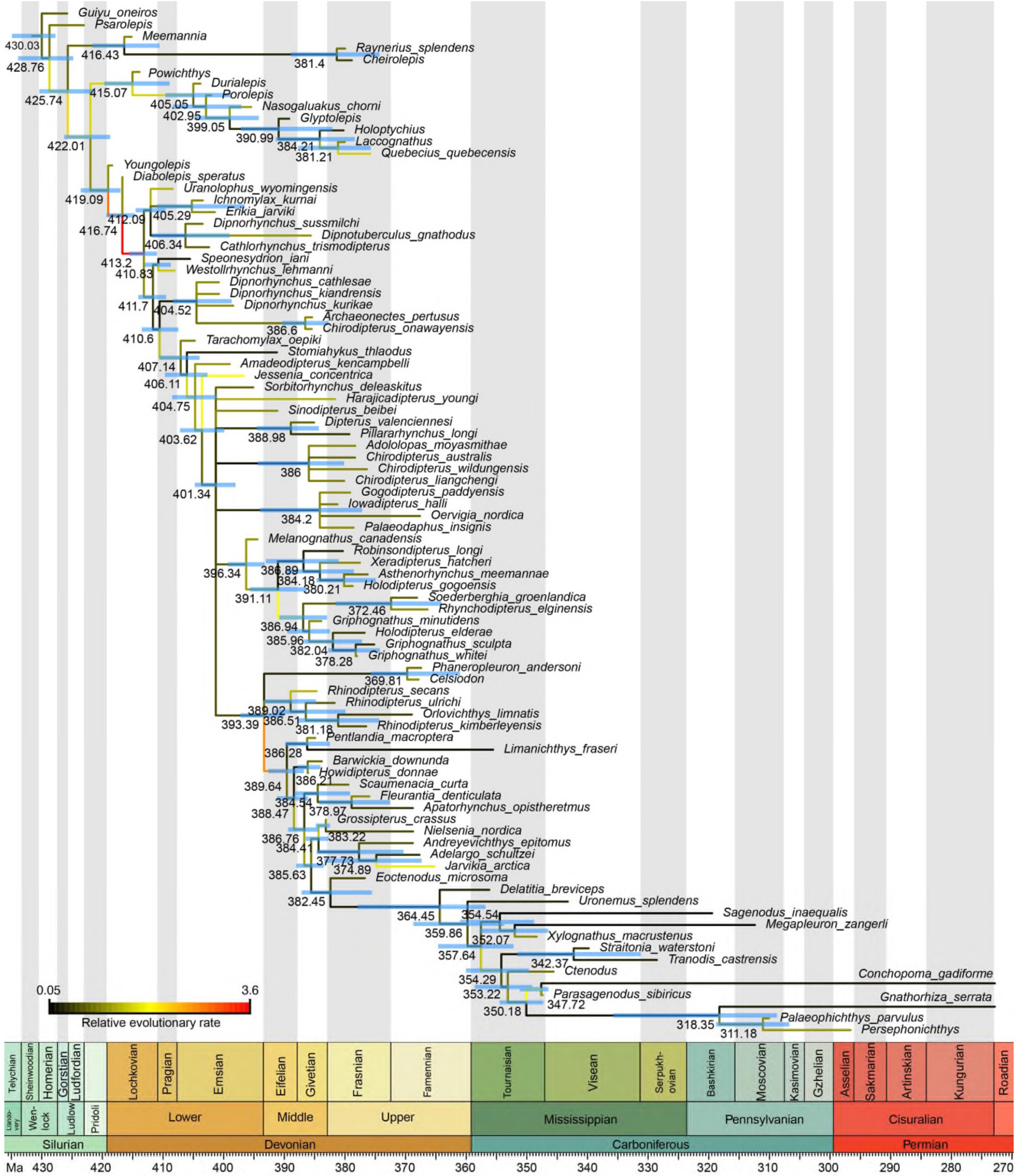

Supplementary Fig. 9 IGR evolutionary rate of characters not associated with the feeding apparatus. The node ages are the posterior medians, with blue bars for each node representing 95% HPD intervals of age estimates. The color of the branch represents the mean relative clock rate at that branch. Partitioned analyses were performed with a topological constraint matching the strict consensus parsimony solution (as in Supplementary Fig. 4).

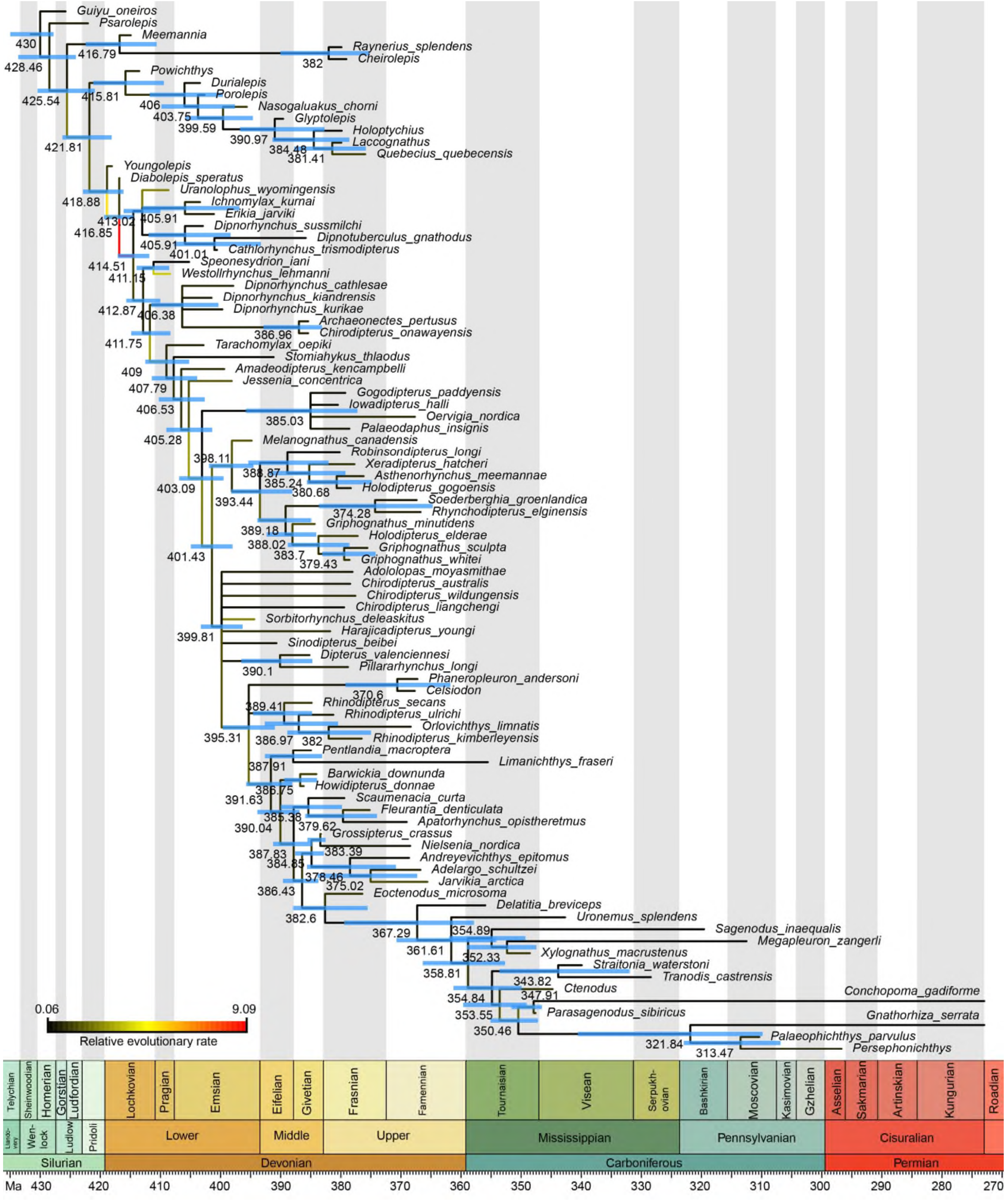

Supplementary Fig. 10 Time-calibrated phylogeny (constrained with strict consensus topology from parsimony analyses) obtained using ILN emphasizing relationships among Palaeozoic Dipnomorpha. The node ages are the posterior medians, with blue bars for each node representing 95% HPD intervals of age estimates. The color of the branch represents the mean relative clock rate along that branch.

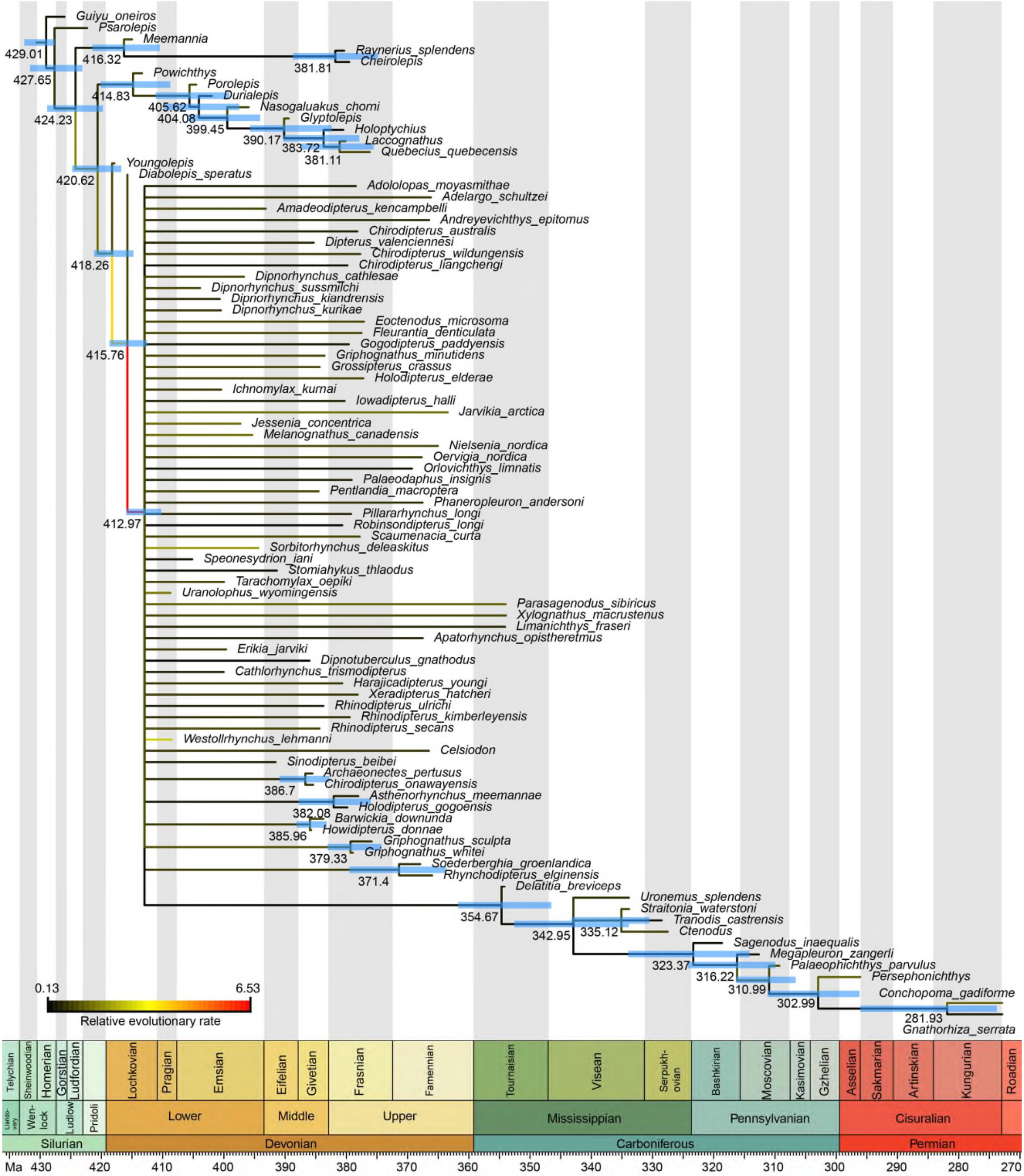

Supplementary Fig. 11 Time-calibrated phylogeny (no topological constraint) obtained using ILN emphasizing relationships among Palaeozoic Dipnomorpha. The node ages are the posterior medians, with blue bars for each node representing 95% HPD intervals of age estimates. The color of the branch represents the mean relative clock rate along that branch.

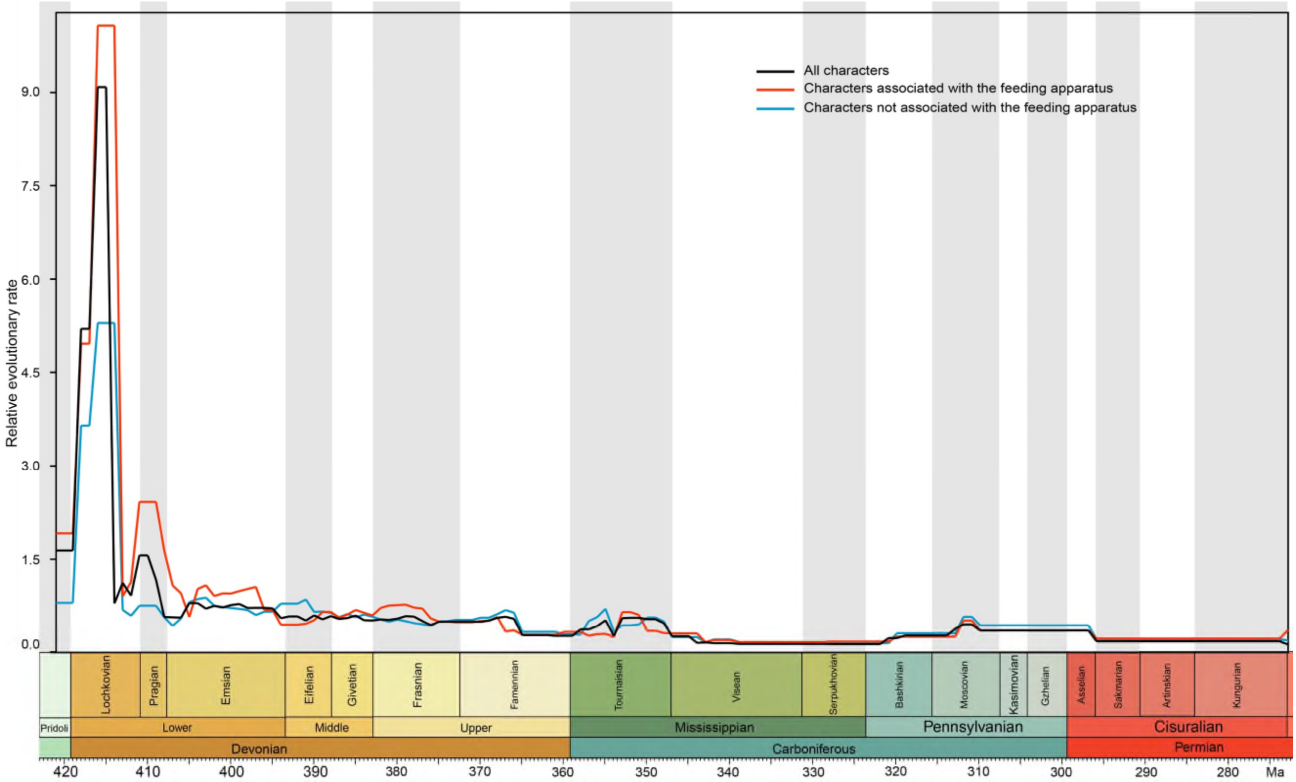

Supplementary Fig. 12 ILN relative rates of phenotypic evolution for all dipnomorphs more closely related to crown lungfishes than to Porolepiformes. Partitioned analyses were performed with a topological constraint matching the strict consensus parsimony solution (as in Supplementary Fig. 10).

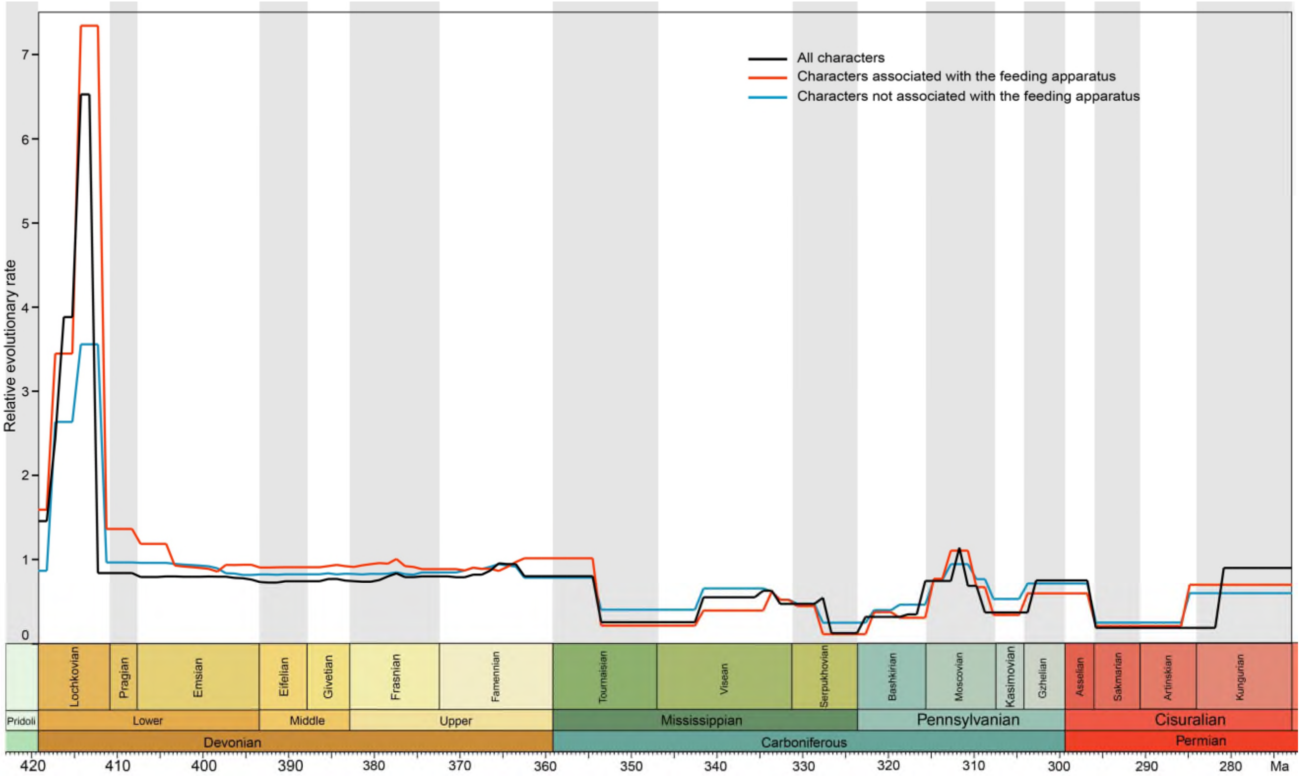

Supplementary Fig. 13 ILN relative rates of phenotypic evolution for all dipnomorphs more closely related to crown lungfishes than to Porolepiformes. Partitioned analyses were performed with no constraint (as in Supplementary Fig. 11).

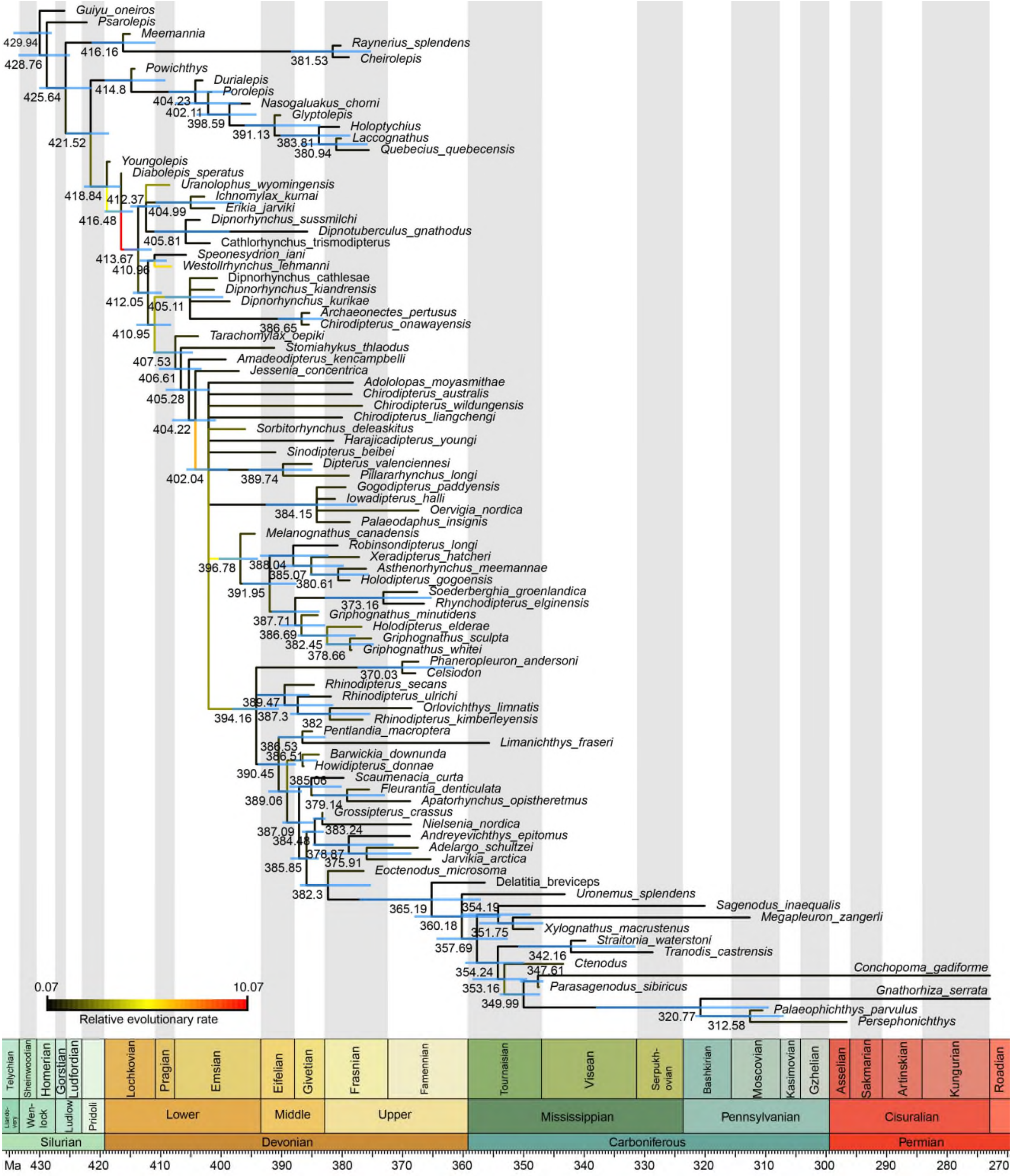

Supplementary Fig. 14 ILN evolutionary rate of characters associated with the feeding apparatus. The node ages are the posterior medians, with blue bars for each node representing 95% HPD intervals of age estimates. The color of the branch represents the mean relative clock rate at that branch. Partitioned analyses were performed with a topological constraint matching the strict consensus parsimony solution (as in Supplementary Fig. 10).

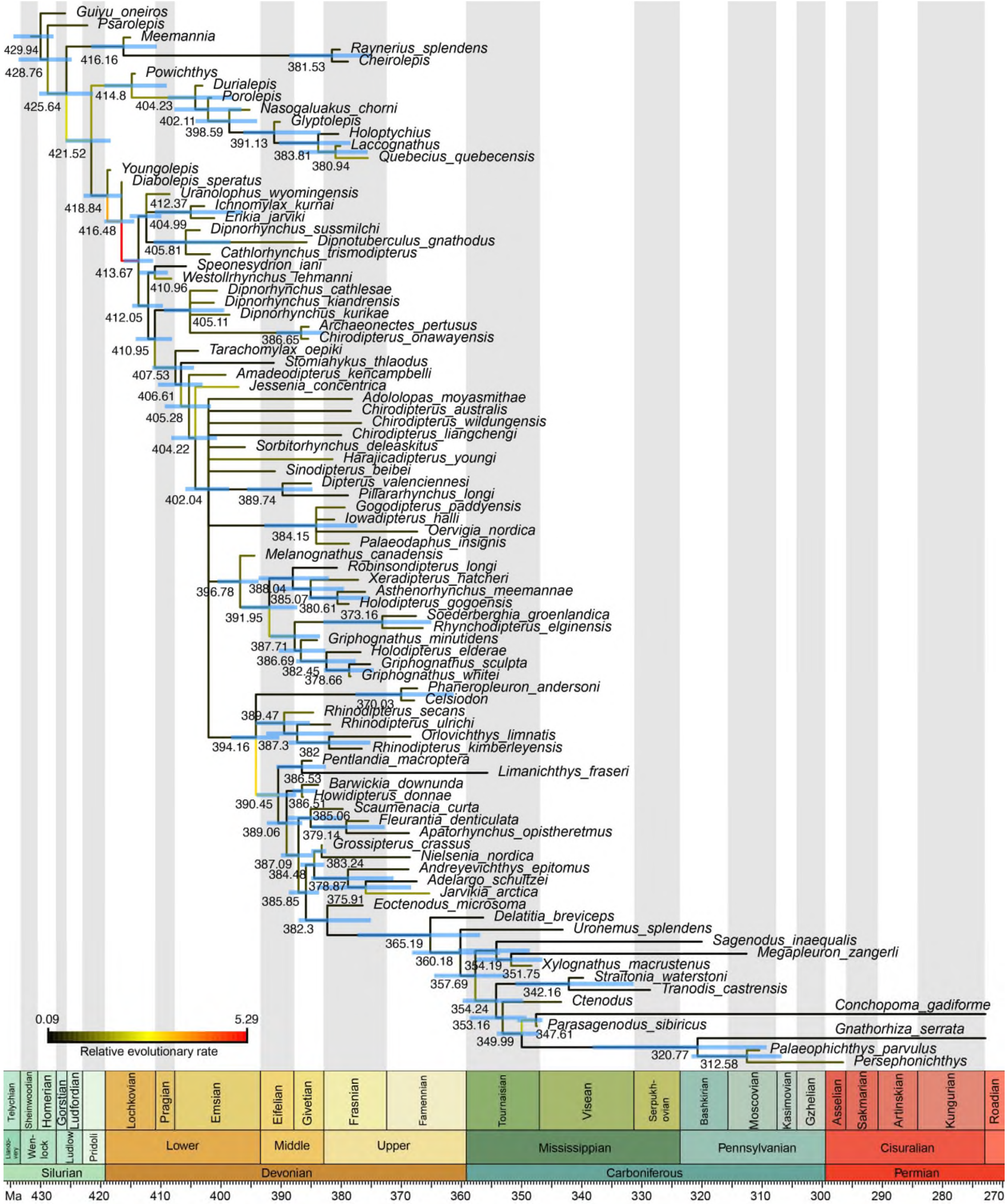

Supplementary Fig. 15 ILN evolutionary rate of characters not associated with the feeding apparatus. The node ages are the posterior medians, with blue bars for each node representing 95% HPD intervals of age estimates. The color of the branch represents the mean relative clock rate at that branch. Partitioned analyses were performed with a topological constraint matching the strict consensus parsimony solution (as in Supplementary Fig. 10).

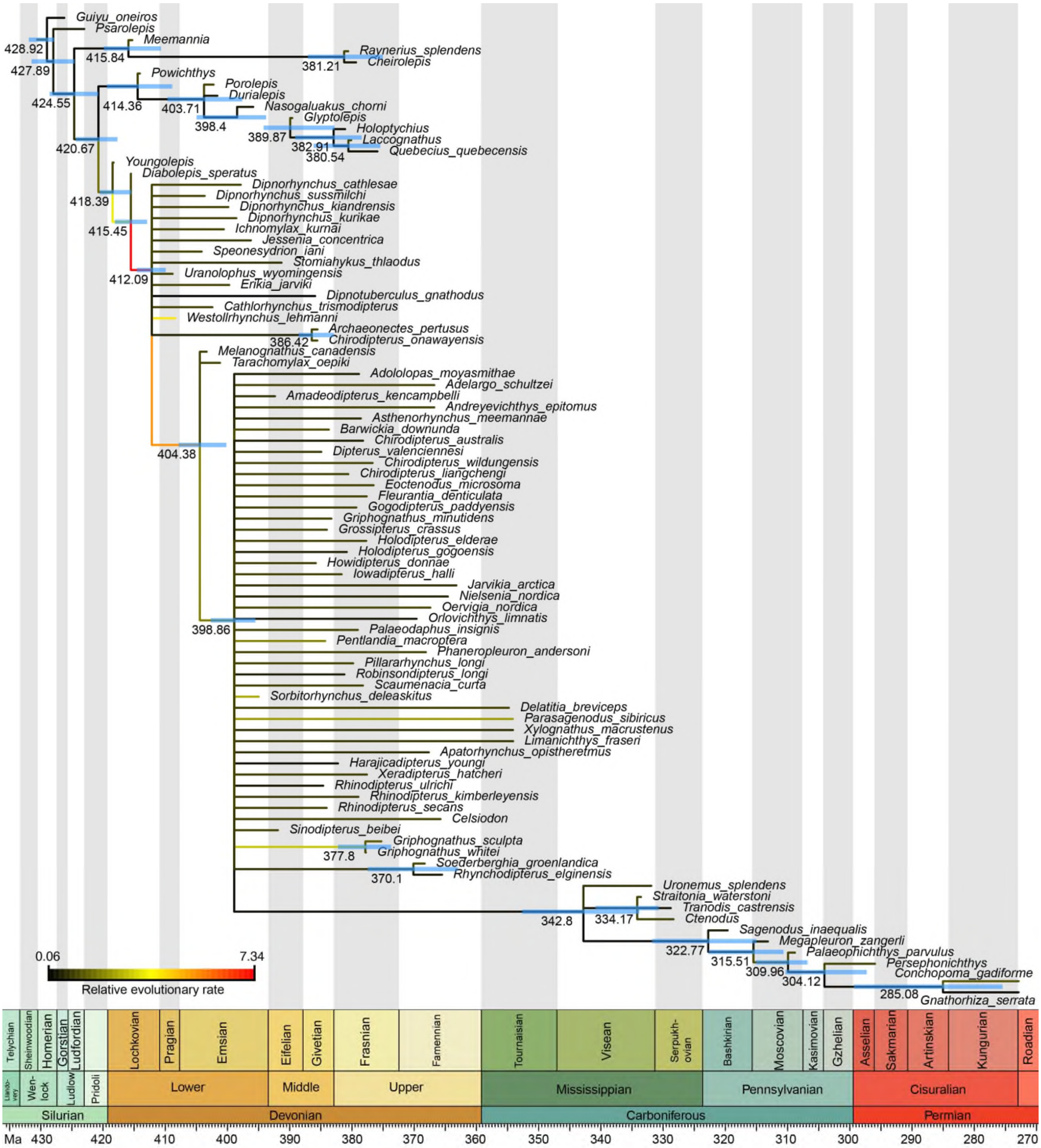

Supplementary Fig. 16 ILN evolutionary rate of characters associated with the feeding apparatus. The node ages are the posterior medians, with blue bars for each node representing 95% HPD intervals of age estimates. The color of the branch represents the mean relative clock rate at that branch. Partitioned analyses were performed with no topological constraint (as in Supplementary Fig. 11).

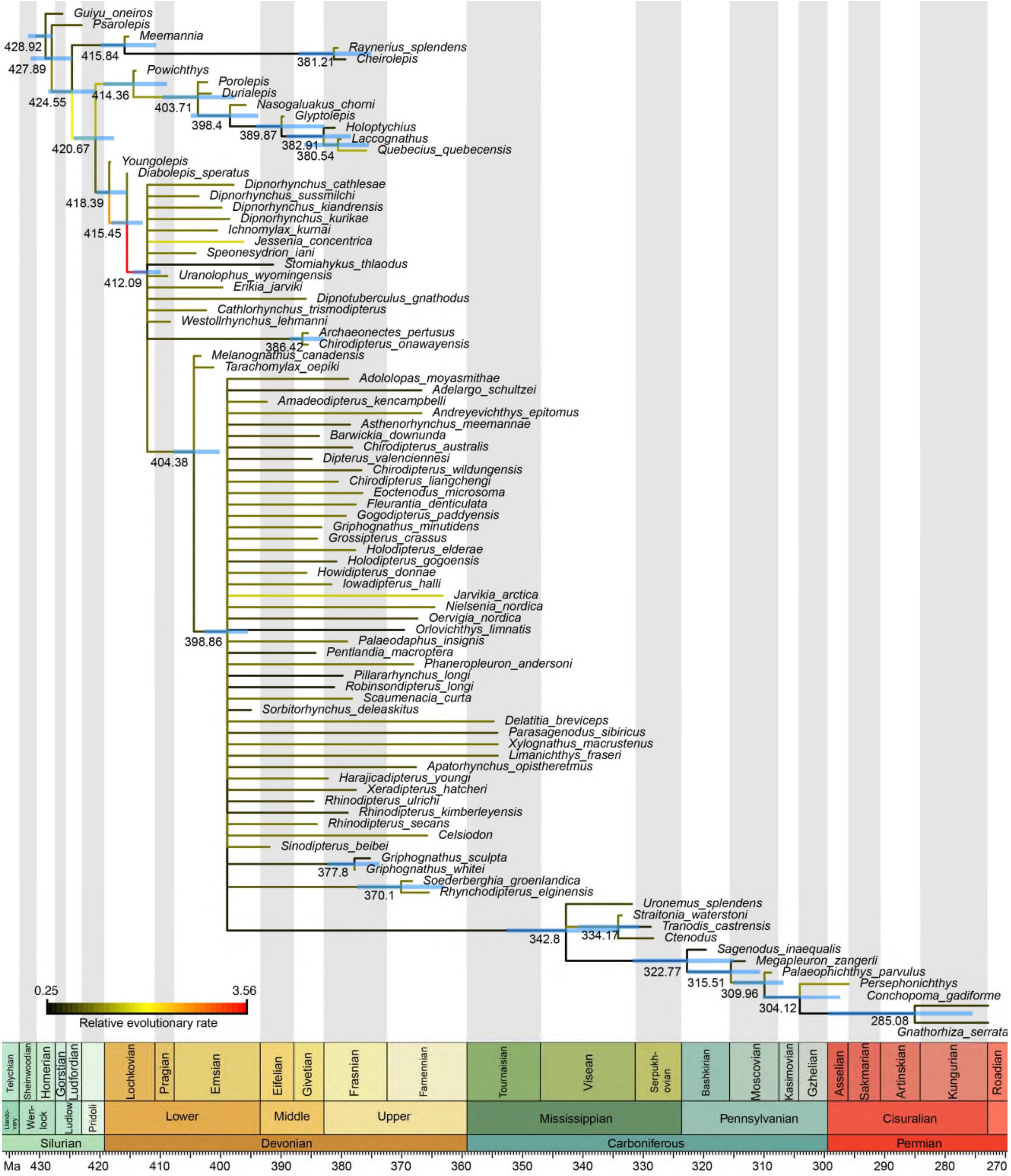

Supplementary Fig. 17 ILN evolutionary rate of characters not associated with the feeding apparatus. The node ages are the posterior medians, with blue bars for each node representing 95% HPD intervals of age estimates. The color of the branch represents the mean relative clock rate at that branch. Partitioned analyses were performed with no topological constraint (as in Supplementary Fig. 11).

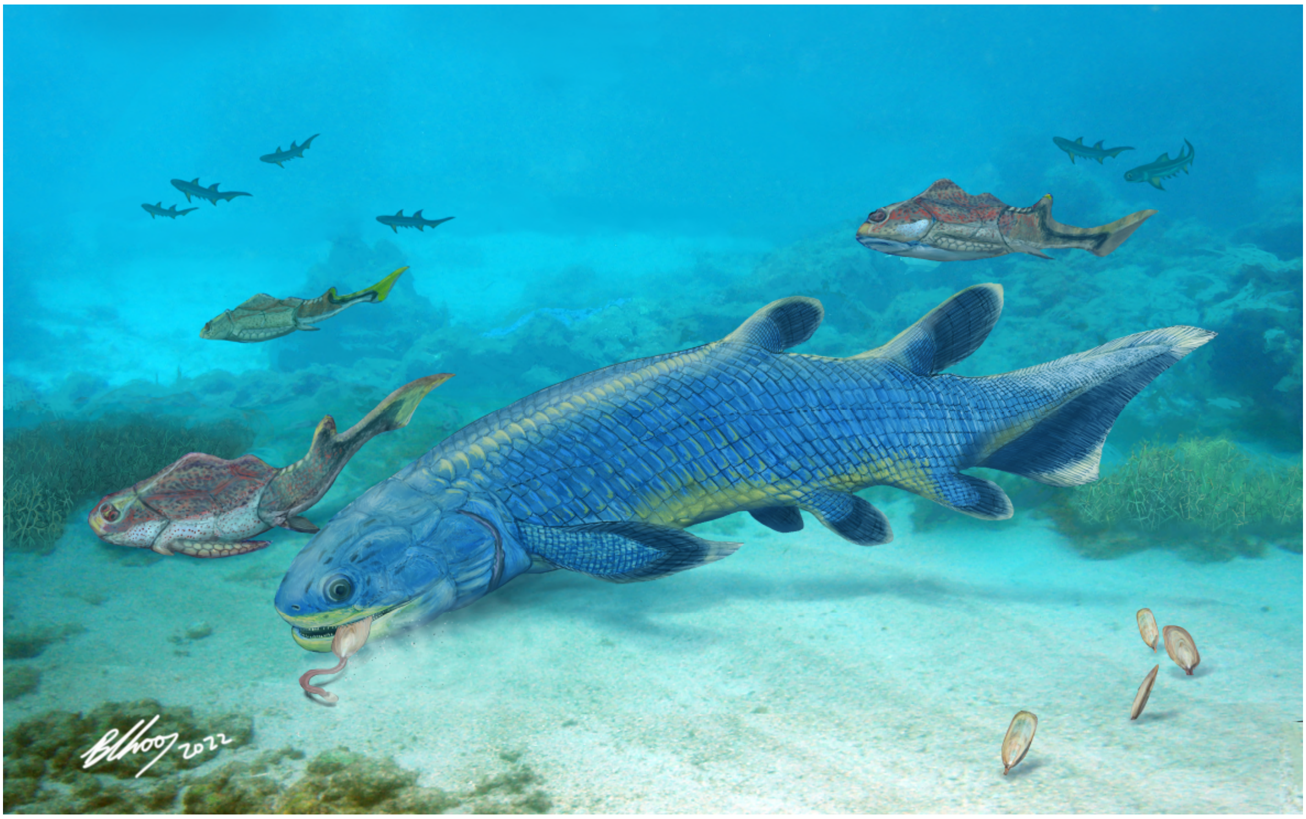

Supplementary Fig. 18 Life reconstruction of *Youngolepis praecursor* and the associated biota. Art credit: Brian Choo.

Supplementary Table

Supplementary Table 1 results of model convergence. Entries in black text indicate converged analyses, while those in grey did not converge. Results illustrated in main text are shown in boldface.

Relaxed Clock	Constraint	Partitions	Convergence (ESS > 200)	Figure
**IGR**	**Yes**	**unpartitioned**	**Yes**	Supplementary Figures 4, 6; Fig. 4
ILN	Yes	unpartitioned	Yes	S Figs. 10, 12
TK02 (autocorrelated rate, AR)	Yes	unpartitioned	No	n/a
IGR	No	unpartitioned	Yes	Supplementary Figs. 5, 7
ILN	No	unpartitioned	Yes	Supplementary Figures 11, 13
TK02 (autocorrelated rate, AR)	No	unpartitioned	No	n/a
**IGR**	**Yes**	**2 partitions**	**Yes**	Supplementary Figures 6, 8, 9; Fig. 4
ILN	Yes	2 partitions	Yes	Supplementary Figures 12, 14, 15
TK02 (autocorrelated rate, AR)	Yes	2 partitions	No	n/a
IGR	No	2 partitions	No	n/a
ILN	No	2 partitions	Yes	Supplementary Figures 13, 16, 17
TK02 (autocorrelated rate, AR)	No	2 partitions	No	n/a

Supplementary Methods

Character list

We assembled a matrix based on characters taken from four analyses: Challands et al.[7] for lungfishes, Schultze[8] for porolepiforms, Lu et al.[9] for sarcopterygians more generally, and Giles et al.[10] for actinopterygians. We also added some new characters, which are indicated below. In cases where taxa have been recoded for specific characters, we note this explicitly.

External dermal bones of the skull

1. **Pineal opening**: 0. open; 1. closed.
2. **Pineal region marked by short eminence**: 0. no; 1. yes. Some early sarcopterygians without pineal foramen in the dermal skull roof bear the pineal region marked by a small, dome-shaped elevation. This structure is found in porolepiforms (*Glyptolepis*, *Holoptychius,* and *Porolepis*)[3] as well as in *Youngolepis*[1] and *Diabolepis*[11]. Friedman[12] argued that this character cannot logically be coded for those taxa that possess a pineal foramen. We therefore change *Guiyu* from '0' to '-', because it has a pineal foramen[13].
3. **Cosmine present on skull**: 0. yes, full cover; 1. yes, but strongly reduced; 2. no. **(Ordered)**
4. **Length of B bone**: 0. short (less than 2 times its width); 1. long (equal or more than 2 times its width); 2. broad (wider than long)
   Diabolepis from '-' to '2' Taxa more distantly related to lungfishes than *Diabolepis* do not possess a B bone[11].
5. **Pit-lines on B bone**: 0. absent; 1. anterior and middle pit-line present; 2. only anterior pit-line; 3. only posterior pit line.
6. **C-bone**: 0. absent; 1. present.
   Psarolepis from '-' to '0'
7. **D-bone**: 0. many; 1. single; 2. absent.
   Psarolepis from '-' to '2'
   Glyptolepis from '0' to '2'
8. **Contact between E and C bones**: 0. absent; 1. present.
9. **Postrostral (Revised)**: 0. absent; 1. postrostral mosaic of small variable bones; 2. large median postrostral, with or without accessory bones; 3. paired E bones; 4. single E-bone.
10. **Length of E-bone(s)**: 0. less than twice their width; 1. more than twice their width.
11. **I-bones meeting in midline**: 0. yes; 1. no, separated by B bone.
    This character changed to just refer to I-bones, which we regard as homologues of the postparietals. In the present matrix, we expanded the ingroup by adding more porolepiforms, which show the primitive contact between the postparietals.
    Psarolepis from '-' to '0'
    Glyptolepis from '-' to '0'
12. **Posterior process of I bone**: 0. absent; 1. present.
    Psarolepis from '-' to '0'
    Youngolepis from '1' to '0'
13. **J-bones (parietals) meeting in midline**: 0. yes; 1. no. This character changed to just refer to J-bones, which we regard as homologues of the parietals.
    Psarolepis form '-' to '0'
14. **L-bone**: 0. two present; 1. one present; 2. fused K+L, 3. fused K+L+M, 4. other bones included.
15. **Length of L-bone**: 0. similar to others in supraorbital canal series; 1. about twice as long as others in supraorbital canal series.
16. **K-bone**: 0. single; 1. space of K+X; 2. neither single nor K+X (e. g. fused (i.e 'space of') K- + L-bones); 3. K-bone absent.
17. **K bone**: 0. medial to X bone; 1. anterior to X bone; 2. in sequence.
18. **M bone**: 0. present; 1. absent.

19. **N bone**: 0. present; 1. absent.
20. **Q bone**: 0. absent; 1. present.
21. **Z bone**: 0. posterior to I bone; 1. lateral to I bone.
22. **Maximum width of skull roof situated posterior to the level of the bone Y_1 (supratemporal)**: 0. yes; 1. no.
    Psarolepis from ‘-’ to ‘0
23. **Sutures between median series of skull roofing bones**: 0. straight; 1. interdigitate; 2. open.
    Powichthys from ‘-’ to ‘0’
24. **Elongated snout**: 0. absent; 1. present.
25. **Ossified upper lip in adult**: 0. mosaic; 1. fused; 2. absent.
    Glyptolepis from ‘0’ to ‘1’
26. **Snout/skull roof**: 0. with diffuse posterior margin; 1. with sharp posterior margin.
27. **Supraorbital and infraorbital canals**: 0. separated; 1. connected.
28. **Lateral line in bone 3**: 0. absent; 1. present.
    Glyptolepis from ‘0’ to ‘-’
    Psarolepis from ‘0’ to ‘-’
29. **Cheek bones**: 0. cheek bones 1–11 present; 1. no 11; 2. no 10, 11.
    Psarolepis from ‘0’ to ‘-’
30. **Length of postorbital cheek**: 0. substantially longer than diameter of orbit; 1. equal to or shorter than diameter of orbit.
31. **Ratio length snout/cheek**: 0. <1; 1. >= 1.
32. **Bone 6**: 0. reaching ventral margin of cheek; 1. excluded from ventral margin of cheek by bone 10.
    Psarolepis from ‘0’ to ‘-’, no bone 6
33. **Bone 7**: 0. approximately equilateral; 1. much longer than deep.
34. **Size of bone 10 (quadratojugal)**: 0. large, as 5(jugal) or greater; 1. much smaller than 5, or absent.
35. **Subopercular**: 0. two; 1. one.
36. **Buccohypophyseal opening (foramen)**: 0. present; 1. absent.
    Psarolepis from ‘?’ to ‘0’.
37. **B-bone (Median skull roof bone between postparietals)**: 0. absent; 1. present.
38. **C-bone(s)**: 0. paired; 1. single. Character state ‘1’ changed from ‘single/absent’ to differentiate between character 6: C-bone: 0. absent; 1. absent.
    Psarolepis from ‘?’ to ‘-’
    Diabolepis from ‘?’ to ‘-’
39. **A-bone (median extrascapular)**: 0. independent A-bone; 1. not present as independent bone; 2. incorporated into skull roof.
    Psarolepis from ‘1’ to ‘?’
40. **F-bone**: 0. not existing; 1. present; 2. place of F+E.
    Psarolepis from ‘?’ to ‘-’
41. **Space taken by K+L or more bones (i.e. K- and L-bones missing if ‘0’)**: 0. not; 1. yes; 2. in addition M; 3. in addition M+N; 4. in addition J+M; 5. in addition X.
42. **G-bone**: 0. present; 1. absent.
43. **I-bone (postparietal)**: 0. present; 1. space of I+J; 2. space of I+J+L+M; 3. space of I+Z; 4. space of A+B+I+J; 5. space of I+Y+Z.
44. **J-bone (parietal)**: 0. present; 1. space of J+K+L+M; 2. space of I+J; 3. space of J+L+M; 4. space of A+B+I+J, 5. space of J+C.
    Psarolepis from ‘?’ to ‘0’
45. **Z-bone (lateral extrascapular)**: 0. behind skull roof; 1. integrated into skull roof; 2. space of Y+Z; 3. lacking as isolated bone. Z-bone is homoglogous with the lateral extrascapular.

46. **Lateral line entering skull table through**: 0. bone Z; 1. bone I; 2. above bones.
Sinodipterus beibei from '?' to '0'
47. **Y-bone**: 0. Y1- and Y2-bones present; 1. only one Y-bone; 2. space of X+Y; 3. space of Y+Z.
48. **X-bone (intertemporal)**: 0. isolated; 1. space of X+K; 2. space of X+Y; 3. missing; 4. fused with the fronto-ethmoidal shield (like *Porolepis* and *Glyptolepis*)[3].
Psarolepis from '?' to '3'
49. **T-bone**: 0. present; 1. absent. T bone only in some lungfishes.
50. **Bone 10 (qudratojugal)**: 0. present, 1. absent.
51. **Bone 11**: 0. Present; 1. absent.
Psarolepis from '?' to '-'
52. **Space taken by L+M**: 0. not present, 1. present, 2. space of J+L+M, 3. space of J+K+L+M (+ possible N), 4. space of I+J+L+M, 5. space of K+L+M.
Psarolepis from '?' to '-'
53. **Tectal**: absent (0); present (1).
54. **Number of tectals**: one (0); two or more (1); absent or no suture (2). Tectals are bones anterior to the orbit that lie between bones carrying the supraorbital canal (nasals) and those carrying the infraorbital canal.
55. **Number of supraorbitals (Revised)**: 0. one (0); 1. two; 2. more than two; 3. missing.
56. **Anterior margin of parietals**: 0. between or in front of orbits (0); 1. slightly posterior to orbits; 2. much posterior to orbits.
57. **Parietal-supraorbital contact**: 0. absent; 1. present.
58. **Supratemporal (Y_1 bone)**: 0. present; 1. absent.
Porolepis from '1' to '0'
Glyptolepis from '1' to '0'
59. **Postparietal and cheek contact**: 0. absent; 1. present.
Porolepis from '1' to '0'
Glyptolepis from '1' to '0'
60. **Dermal joint between parietal and postparietal**: 0. absent;1. present.
61. **Processus dermintermedius**: 0. absent; 1. present. This is a process of dermal bone that extends into the opening of the nostril. It is very hard to determine presence or absence.
62. **Ethmoid commissure (sensory canal)**: 0. present; 1. absent.
63. **Course of ethmoid commissure**: 0. middle portion through median rostral; 1. sutural course; 2. through bone centre.
64. **Relationship of infraorbital canal to premaxilla**: 0. infraorbital canal entering premaxilla; 1. infraorbital canal following dorsal margin of premaxilla.
65. **Course of otic sensory canal**: 0. not through growth centre of postparietal; 1. through growth centre of postparietal.
66. **Otic canal extends through postparietals**: 0. Absent; 1. Present.
67. **Posterior end of supraorbital sensory canal**: 0. in postparietal; 1. in parietal; 2. in intertemporal.
68. **Otic and supraorbital sensory canals**: 0. not in contact; 1. in contact.
69. **Position of posterior pit line**: 0. on posterior half of postparietal; 1. on anterior half of postparietal.
70. **Shape of jugal**: 0. short and deep; 1. long and low.
71. **Prespiracular**: 0. absent; 1. present.
72. **Squamosal, quadratojugal and preopercular bones**: 0. Separated; 1. fused.
73. **Subsquamosals**: 0. absent; 1. present.
74. **Preoperculosubmandibular**: 0. absent; 1. present.
75. **Foramina on dermal cheek bones**: 0. absent; 1. present.
76. **Vertical bar-like preopercular bone**: 0. absent; 1. present.

77. **Postorbital**: 0. restricted to anterior cranial division; 1. spanning two divisions.
78. **Median gular**: 0. present; 1. absent.
79. **Sensory line network**: 0. preserved as open grooves (sulci) in dermal bones; 1. sensory lines pass through canals in dermal bones (open as pores).
80. **Westoll lines**: 0. absent; 1. present.
81. **Canal-bearing bone of skull roof extends far past posterior margin of parietals**: 0. no, 1. yes.

Oral elements (palate part)

82. **Palatal construction**: 0. parasphenoid separates pterygoids; 1. pterygoids articulate with each other with suture; 2. pterygoids fused.
83. **Parasphenoid**: 0. fused into palate; 1. visible sutures; 2. overlapping (pterygoids).
84. **Transverse curvature of palate**: 0. flat; 1. arched.
85. **Parasphenoid stalk**: 0. no stalk; 1. simple stalk without sharp division into tapering proximal portion and parallel-sided distal portion; 2. stalk with sharp division into tapering proximal portion and parallel-sided distal portion. **(Ordered)**.
86. **Ratio of posterior length to anterior length of parasphenoid**: 0. less than 1 or about 1; 1. greater than 1. This distinction is usually made between the body (or corpus) of the parasphenoid and the stalk. In some taxa this distinction is very clear. But in many cases, this is not so obvious (e.g. *Dipterus*).
87. **Furrow on ventral surface of parasphenoid stalk**: 0. absent; 1. present.
    Psarolepis from ‘0’ to ‘-’
88. **Furrow on dorsal surface of parasphenoid stalk**: 0. absent; 1. present.
89. **Parasphenoid bearing denticle-lined ascending process**: 0. no; 1. yes.
    Chirodipterus wildungensis from ‘?’ to ‘0’
90. **Dental material on parasphenoid**: 0. present; 1. absent.
    Psarolepis from ‘?’ to ‘0’
91. **Parasphenoid reaching posterior margin of occiput**: 0. no; 1. yes. This assumes the presence of a stalk. Can only be coded for taxa with a stalked paraphenoid.
    Chirodipterus wildungensis from ‘?’ to ‘1’
92. **Shape of parasphenoid**: 0. anteriad elongated, 1. plow-shaped, 2. with lozenge, 3. round anterior portion, 4. angled anterior portion.
93. **Position of parasphenoid**: 0. below ethmosphenoid; 1. below otico-occipital; 2. below both.
94. **Position of anterior end of parasphenoid**: 0. in front of jaw articulation; 1. not in front.
95. **Ratio of the maximum width of parasphenoid to the distance of articulation points of jaws**: 0. less than 1/3; 1. between 1/3 and 2/3; 2. greater than 2/3.
96. **Lateral angle of parasphenoid**: 0. no angle, 1. angular, 2. rounded, 3. reflexed.
    Psarolepis from ‘-’ to ‘0’
97. **(Posterior) end of parasphenoid (stalk)**: 0. single point; 1. bifid; 2. trifid with lateral projections.
    Chirodipterus wildungensis from ‘?’ to ‘1’
98. **Margins of posterior stalk of parasphenoid**: 0. converge to posterior angle; 1. subparallel.
    Psarolepis from ‘0’ to ‘-’
    Chirodipterus wildungensis from ‘?’ to ‘0’
99. “Vomer” sensu Miles (1977): 0. present; 1. absent.
    Chirodipterus wildungensis from ‘?’ to ‘0’
100. **Vomer**: 0. Paired; 1. Unpaired.
101. **“Dermopalatine 1”**: 0. median; 1. paired.
102. **“Dermopalatine 1” / pterygoid**: 0. fused to pterygoid; 1. sutured to pterygoid; 2. isolated.
    Psarolepis from ‘1’ to ‘?’

103. **Series anterolateral to pterygoids (dermopalatine, extopterygoid, and vomer)**: 0. present, with tusks; 1. present with denticles or dentine sheet; 2. present with tooth row. This is interpreted as meaning ‘dental’ series anterolateral to pterygoids. Here, we consider the series anterolateral to pterygoids include the dermopalatine, extopterygoid, and vomer.
Chirodipterus wildungensis from ‘?’ to ‘1’
104. **Parasphenoid separating pterygoids along more than half of their length**: 0. yes; 1. no.
Chirodipterus wildungensis from ‘?’ to ‘1’
105. **Angle between midline and anterolateral margin of pterygoid**: 0. less than 55 degrees; 1. more than 55 degrees.
Chirodipterus wildungensis from ‘?’ to ‘1’
106. **Cosmine-like tissue within oral cavity**: 0. no; 1. yes.
107. **Angle between quadrate and plane of parasphenoid**: 0. 90–95 degrees; 1. 80–65 degrees; 2. 55–35 degrees.
Psarolepis form ‘0’ to ‘?’
108. **Autostyly**: 0. absent; 1. present.
109. **Lateral commissure**: 0. separate from palatoquadrate; 1. partly fused but distinguishable; 2. wholly fused to palatoquadrate. **(Ordered)**.
Chirodipterus wildungensis from ‘?’ to ‘2’
110. **Palatoquadrate**: 0. fused into palate; 1. free.
Psarolepis from ‘1’ to ‘?’
Chirodipterus wildungensis from ‘?’ to ‘0’
111. **Dorsolateral process on palatoquadrate**: 0. absent; 1. present.
112. **Overlap relationship between entopterygoids and parasphenoid**: 0. parasphenoid overlaps entopterygoids dorsally; 1. entopterygoids overlap parasphenoid dorsally.
Guiyu from ‘0’ to ‘-’
Chirodipterus wildungensis from ‘?’ to ‘0’
113. **Median callus on palate**: 0. absent; 1. present.
Youngolepis from ‘?’ to ‘0’
Chirodipterus wildungensis from ‘?’ to ‘0’
114. **Articulation of parasphenoid**: 0. parasphenoid not sutured to vomer; 1. parasphenoid sutured to vomer.
115. **Palatoquadrate fused with neurocranium**: 0. absent; 1. present.
116. **Entopterygoids**: 0. separated; 1. contact along midline.

Neurocranium (Nasal part)
117. **Anterior nostril**: 0. located dorsal to oral margin; 1. marginal.
Chirodipterus wildungensis from ‘?’ to ‘1’
118. **Posterior nostril**: 0. located dorsal to oral margin; 1. marginal 2. palatal. **(Ordered)**.
Youngolepis from ‘1’ to ‘0’
119. **Internasal pits**: 0. well developed; 1. reduced or absent.
Chirodipterus wildungensis from ‘?’ to ‘1’
120. **Pore cluster**: 0. absent; 1. present.
121. **Rostral tubuli (tubules)**: 0. absent; 1. present.
Gogonasus from ‘?’ to ‘1’
122. **Size of profundus canal in postnasal wall**: 0. small; 1. large.

Oral elements (Dentition and jaw bones)
123. **Premaxilla**: 0. present; 1. absent.
124. **Maxilla**: 0. absent, 1. present.
125. **Lateral lines in mandible**: 0. parallel; 1. converging in one bone.

Holoptychius from ‘?’ to ‘1’

126. **Length of symphysis (ratio length of symphysis to length of jaw)**: 0. greater than 1/3; 1. between 1/5 and 1/3; 2. less than 1/5.

127. **Adsymphysial plate (MdY bone)**: 0. present, but fused, 1. isolated, sutured bone, 2. missing. Here we consider the adsymphysial is homologized with the parasymphysial dental plate in osteolepiforms, porolepiforms.

128. **Parasymphysial tooth whorl**: 0. tooth whorl; 1. denticles; 2. plate with a tusk. New character. The parasymphysial and the adsymphysial are homologous. And they all belong to the coronoid series.

129. **“Dentary”**: 0. unpaired; 1. paired; 2. absent. Some lungfishes have the unpaired ‘dentary’ between the left and right lower jaws.
Dipnorhynchus kurikae from ‘?’ to ‘0’

130. **Dentary-prearticular relationship**: 0. dentition-generating gap; 1. small midline hole only; 2. no gap.

131. **Slot between dentary and prearticular**: 0. broad; 1. narrow; 2. no slot.

132. **Adductor fossa**: 0. not overhung by prearticular; 1. overhung by prearticular.
Psarolepis from ‘1’ to ‘0’
Dipnorhynchus kurikae from ‘?’ to ‘0’
Griphognathus sculpta from ‘?’ to ‘1’
Griphognathus whitei from ‘?’ to ‘1’

133. **Length of adductor fossa (revised)**: More than 1/3; 1. 20%–1/3 of jaw length; 2. 5–20% of jaw length; 3. 0–5% of jaw length.
Chirodipterus wildungensis from ‘?’ to ‘1’
Dipnorhynchus kurikae from ‘?’ to ‘0’
Griphognathus sculpta from ‘?’ to ‘3’
Griphognathus whitei from ‘?’ to ‘3’

134. **Morphology of adductor fossa**: 0. open; 1. reduced to vestigial slit.
Dipnorhynchus kurikae from ‘?’ to ‘0’
Griphognathus sculpta from ‘?’ to ‘1’
Griphognathus whitei from ‘?’ to ‘1’

135. **Coronoids**: 0. present; 1. absent.
Dipnorhynchus kurikae from ‘?’ to ‘1’

136. **Lip fold**: 0. absent; 1. present.
Dipnorhynchus kurikae from ‘?’ to ‘1’
Griphognathus sculpta from ‘?’ to ‘1’
Griphognathus whitei from ‘?’ to ‘1’

137. **Meckelian bone**: 0. wholly ossified; 1. only articular ossified, or not ossified at all.
Griphognathus whitei from ‘?’ to ‘0’

138. **Retroarticular process**: 0. small and poorly developed; 1. robust, squarish.
Griphognathus whitei from ‘?’ to ‘1’

139. **Skin contact surface on infradentary bones**: 0. reaching up to lip of adductor fossa; 1. widely separated from lip of adductor fossa.
Griphognathus sculpta from ‘?’ to ‘1’
Griphognathus whitei from ‘?’ to ‘1’

140. **Curvature of ventral mandibular margin**: 0. strongly convex; 1. essentially flat.
Griphognathus sculpta from ‘?’ to ‘1’
Griphognathus whitei from ‘?’ to ‘1’

141. **Orientation of glenoid**: 0. mostly dorsally; 1. posterodorsally.
Griphognathus sculpta from ‘?’ to ‘1’
Griphognathus whitei from ‘?’ to ‘1’

142. **Shape of glenoid fossa**: 0. double structure; 1. single groove.
Griphognathus sculpta from ‘?’ to ‘0’
Griphognathus whitei from ‘?’ to ‘1’
143. **Angular (infradentary 3) and surangular (infradentary 4)**: 0. separate; 1. fused into a single long bone.
144. **Splenial (infradentary 1) and postsplenial (infradentary 2)**: 0. separate; 1. fused.
145. **Teeth on upper lip**: 0. shedding teeth; 1. statodont tooth row; 2. teeth absent.
146. **Teeth on dentary**: 0. shedding teeth present; 1. statodont tooth rows present; 2. teeth absent.
147. **Number of tooth ridges in adult specimens**: 0. <10; 1. >10.
Psarolepis from ‘0’ to ‘?’.
148. **Lower jaw**: 0. short mandible rami (most lungfishes), 1. elongated rami with short symphysis (most outgroup), 2. elongated symphysis.
Diabolepis from ‘?’ to ‘1’
Dipnorhynchus kurikae from ‘?’ to ‘0’
149. **Adductor muscles**: 0. below skull roof, 1. above skull roof.
150. **Number of infradentaries**: 0. four, 1. two, 2. one, 3. three.
151. **Ossified Meckelian bone**: 0. present, 1. lacking.
152. **Posteriorly deep maxilla**: 0. present; 1. absent.
Youngolepis from ‘0’to ‘1’
153. **Anterior end of dentary**: 0. not modified; 1. modified into support for parasymphysial tooth whorl.
154. **Axis of parasymphysial tooth whorl**: 0. parallel to dentary; 1. perpendicular to dentary.
Holoptychius from ‘1’ to ‘0’
155. **Dentition on coronoid**: 0. broad marginal “tooth field”; 1. narrow marginal tooth row; 2. single tooth row.
156. **Foramina on external surface of lower jaw**: 0. absent; 1. present.
157. **Length of dentary**: 0. constitutes a majority of jaw length 1. half the length of jaw or less.
158. **Labial pit**: 0. absent; 1. Present.
159. **Prearticular symphysis**: 0. absent; 1. present.
160. **Inturned medial process of premaxilla**: 0. absent; 1. present.
161. **Coronoids**: 0. Four or more; 1. Three or less.
162. **Premaxilla**: 0. Extends under orbit; 1. Restricted anterior to orbit.
163. **Maxilla shape**: 0. Splint-shaped; 1. Cleaver-shaped.

Oral elements (Tooth plate)
164. **Tooth plates**: 0. present; 1. absent.
Chirodipterus wildungensis from ‘?’ to ‘1’
165. **Morphology of teeth on pterygoid and prearticular**: 0. round/conical; 1. forming distinct proximodistal cutting ridge.
166. **Addition of large dentine elements at regular intervals to lateral margin of pterygoid/ prearticular**: 0. yes; 1. no.
167. **Nature of large dentine elements**: 0. teeth; 1. petrodentine cores; 2. thick irregular dentine; 3. ridges narrow regular dentine ridges.
168. **Addition of marginal blisters to pterygoid/prearticular**: 0. no; 1. yes.
169. **Shape of marginal blisters**: 0. bead-shaped; 1. elongated strips.
170. **Addition of inter-row dentine along edge of pterygoid/prearticular**: 0. no; 1. yes.
171. **Nature of inter-row dentine**: 0. always fuses or wears down into sheet; 1. separate denticles persist between some tooth rows.
172. **Pulp cavity**: 0. tooth plates without pulp cavity; 1. with pulp cavity.
173. **Diffuse dentine deposition on surface of palate/lower jaw**: 0. yes, diffusely across whole

palate; 1. no; 2. redeposition of denticles only within "footprint" (outer circumference) of resorbed tooth plate.

174. **Relative areas of denticle field/thin dentine sheet on palate**: 0. all or nearly all denticles; 1. both dentine sheet and denticles; 2. mostly dentine sheet; 3. denticles outside tooth plate; 4 dentine sheet on resorption areas within tooth plate.
 Youngolepis from '0' to '3'
175. **Relative areas of denticle field and dentine sheet on lower jaw**: 0. all or nearly all denticles; 1. both denticles and dentine sheet; 2. mostly dentine sheet.
176. **Resorption of dentition on pterygoid/prearticular plate origin**: 0. little or no resorption, origin left unmodified; 1. extensive resorption, removing mesial parts of plate; 2. resorption and deposition of dentine sheet within toothplate only, not crossing edges.
 Youngolepis from '?' to '0'
177. **Distinct vertically growing "heel" on prearticular**: 0. no; 1. yes.
178. **Petrodentine**: 0. absent; 1. present. Petrodentine is a highly mineralized tissue noticeably analogous to that of enameloid, and is deposited intermittently in a proximal direction by the sole participation of mesenchymal petroblasts.
179. **Sharp "additive" mesial and posterior edges on tooth plates**: 0. absent; 1. present.
 Psarolepis from '0' to '?'
180. **Behaviour of "additive edges" (if present)**: 0. quiescent; 1. active.
181. **Angle between first and last tooth ridge**: 0. 50–100du° ; 1. less than 50° or greater than 100° .
 Youngolepis from '-' to '0'
182. **Ascending process on pterygoid**: 0. absent; 1. short; 2. long.
183. **Dentition on the entoptergoid**: 0. dentine plates; 1. tooth plates; 2. toothed (shedding denticles).
 Youngolepis from '1/2' to '1'
 Psarolepis from '2' to '-'
184. **Form of marginal tooth ridge**: 0. absent; 1. continuous; 2. incomplete.
 Psarolepis from '0' to '?'
185. **Tuberosities (denticles) on palate**: 0. present and irregular; 1. arranged radially; 2. arranged in rows; 3. absent.
186. **Denticles**: 0. no denticles; 1. episodically shed denticles.
187. **Tooth plates ridges**: 0. no tooth plates; 1. without radial pattern; 2. radial pattern with cusps; 3. radial pattern without cusps; 4. parallel ridges.

Neurocranium

188. **Braincase/skull table relationship**: 0. broad contact; 1. supported by cristae.
189. **Metotic (lateral otic) fissure**: 0. present; 1. absent.
190. **Intracranial joint/ventral cranial fissure**: 0. mobile joint; 1. ventral cranial fissure; 2. neither fissure nor joint.
 Dipnorhynchus kurikae from '?' to '2'
191. **Occiput inset from posterior margin of neurocranium**: 0. no; 1. yes.
192. **Notochordal canal occluded by ossified cranial centrum**: 0. no; 1. yes.
193. **Neural cavity and notochordal canal separated by an ossified shelf in the occipital region, posterior to the foramen for N. X**: 0. yes; 1. no.
194. **Ossification complete along ventral midline of notochordal canal posteriorly**: 0. yes; 1. no.
195. **Occipital region bears transverse processes flanking foramen magnum**: 0. no; 1. yes.
196. **Dorsal aorta**: 0. divides at or anterior to occiput; 1. divides posterior to occiput.
197. **Lateral dorsal aortae**: 0. run along ventral surface of neurocranium; 1. run in grooves on parasphenoid.

198. **Occipital artery extramural**: 0. no; 1. yes.
199. **Neurocranium extends far posterior to hind margin of postparietals**: 0. no; 1. yes.
200. **Dorsolateral crista fenestrated**: 0. no; 1. yes.
201. **Median crista discontinuous**: 0. no; 1. yes.
202. **Little or no overlap between intersections of median and dorsolateral cristae with the dermal skull roof (median crista abbreviated)**: 0. no; 1. yes.
203. **Lateral cristae fenestrated**: 0. no; 1. yes.
204. **Development of a pronounced ridge anterior to and continuous with the dorsolateral cristae**: 0. no; 1. yes.
    Psarolepis from '0' to '-'
205. **Articulation of first epibranchial posterior to the level of the foramen for N. IX**: 0. no; 1. yes.
206. **Notochord extending to or beyond level of N. V**: 0. yes; 1. no.
    Guiyu from '?' to '0'
    Psarolepis from '?' to '0'
207. **Development of a deep "spiracular recess"**: 0. yes; 1. no.
    Psarolepis from '0' to '-'
208. **Separate foramina for the internal carotid artery and efferent pseudobranchial artery**: 0. no; 1. yes.
209. **Jugular vein**: 0. little or no groove; 1. travels through deep groove along length of otic region.
210. **Foramina for the jugular vein and the ramus hyomandibularis N. VII on the posterior surface of the transverse wall of the otic region**: 0. confluent; 1. separate.
211. **Foramina for the jugular vein and the orbital artery on the posterior surface of the transverse wall of the otic region**: 0. confluent; 1. separate.
212. **Foramina for the ramus hyomandibularis N. VII and the orbital artery on the posterior surface of the transverse wall of the otic region**: 0. confluent; 1. separate.
213. **Hyomandibular facet tranverses fissure in transverse otic wall (hyomandibular facet extends on to palatoquadrate)**: 0. no; 1. yes.
    Gogonasus from '?' to '0'
    Glyptolepis from '?' to '0'
    Youngolepis from '?' to '0'
    Powichthys from '?' to '0'
    Diabolepis from '?' to '0'
214. **Separate ossified canals for pineal and parapineal organs**: 0. yes; 1. no.
    Psarolepis from '0' to '?'
215. **Foramen for N. II above the level of foramen sphenoticum minus**: 0. no; 1. yes.
216. **Foramen for N. III above level of foramen sphenoticum minus**: 0. no; 1. yes.
217. **Ventral face of nasal capsule**: 0. complete; 1. perforated by fenestration that opens posteroventrolaterally (fenestra ventralis); 2. solum nasi completely unossified. **(ordered)**.
218. **Nasal capsule set well posterior to snout margin or preoral eminence**: 0. no; 1. yes.
219. **Enlarged, knob-shaped protrusion on the posteroventral surface of the quadrate (hyosuspensory eminence)**: 0. absent; 1. present.
220. **Adlateral cristae (posterodorsal extensions of the lateral cristae that connect the otic region of the neurocranium to the visceral surface of the dermal skull roof) present**: 0. yes; 1. no.
221. **Foramen for the internal carotid anterior to that for the efferent pseudobranchial artery**: 0. no; 1. yes.
222. **Ossification of neurocranium**: 0. completely ossified; 1. poorly-ossified/cartilaginous.
223. **Kinesis between nasal region and braincase behind it**: 0. absent; 1. present.
224. **Supraoccipital commissure**: 0. through Z-G-I-A-I-G-Z; 1. through I-A-I; 2. through I-B-I; 3. through Z-B-Z; 4. above bones.

225.**Buccohypophysial foramen of parasphenoid**: 0. single; 1. double.
Diabolepis from ‘?’ to ‘0’
226.**Parasphenoid**: 0. protruding forward in ethmoid region of endocranium; 1. behind ethmoid region.
227.**Dorsal endoskeletal articulation between otico-occipital and ethmosphenoid blocks of braincase**: 0. absent; 1. present.
228.**Ventral endoskeletal articulation between otico-occipital and ethmosphenoid blocks of braincase**: 0. absent; 1. present.
229.**Orientation of intracranial joint or fissure**: 0. vertical or anteroventrally slanting; 1. posteroventrally slanting.
230.**Position of intracranial joint or fissure relative to cranial nerves**: 0. joint through profundus foramen; 1. joint through trigeminal foramen.
231.**Processus descendens of sphenoid**: 0. absent; 1. present.
232.**Fossa autopalatina**: 0. absent; 1. present.
233.**Vomeral area with grooves and raised areas**: 0. absent; 1. present.
Psarolepis from ‘1’ to ‘0’
234.**Fenestra ventralis**: 0. absent; 1. large, medially situated; 2. common ventral fenestra for anterior and posterior nostrils.
235.**Large median opening and several small dorsolateral openings in postnasal wall**: 0. absent; 1. present.
236.**Postorbital process on braincase**: 0. present; 1. absent.
237.**Basicranial fenestra with arcual plates**: 0. absent; 1. present.
238.**Otico-sphenoid bridge**: 0. present; 1. absent.
239.**Posttemporal fossae**: 0. absent; 1. present.
240.**Prominent pre-orbital rostral expansion of the neurocranium**: 0. present; 1. absent.
241.**Spiracular groove on basicranial surface**: 0. absent; 1. Present.
242.**Endoskeletal spiracular canal**: 0. open; 1. partial enclosure or spiracular bar; 2. complete enclosure in canal.
243.**Entrance of internal carotids**: 0. through separate openings flanking the hypophyseal opening or recess; 1. through a common opening at the central midline of the basicranium.
244.**Articulation between neurocranium and palatoquadrate posterodorsal to orbit (suprapterygoid articulation)**: 0. absent 1. present.
245.**Vestibular fontanelle**: 0. absent 1. present.
Porolepis from ‘?’ to ‘1’
246.**Paired pineal and parapineal tracts**: 0. absent; 1. present.
247.**Lateral cranial canal**: 0. absent, 1. present.

Postcranium (Fins and girdles)
248.**Cleithrum and clavicle**: 0. with cosmine; 1. without cosmine.
Psarolepis from ‘?’ to ‘0’
249.**Median fin morphologies**: 0. all separate and short-based; 1. posterior dorsal fin long-based; 2. both dorsal fins long-based uninterrupted fin fringe.
250.**Posterior dorsal fin support**: 0. all radials carried by basal plate; 1. anterior radials on basal plate, posterior radials free; 2. no basal plate.
251.**Anal fin support**: 0. trapezoidal with no distinct shaft; 1. cylindrical proximal shaft and triangular distal plate.
252.**Median fin radials**: 0. cylindrical; 1. hourglass-shaped.
253.**Dorsal cleithrum (AL of the Placodermi), ventral cleithruin (AVL of the Placodermi) and pectoral spine (SP of the Placodermi)**: 0. not fused; 1. fused.
254.**Interclavicle**: 0. absent; 1. present.

255. **Proximal articular surface of humerus**: 0. concave; 1. flat: convex.
256. **Basal plates in dorsal fin supports**: 0. absent; 1. present.
257. **Shape of dorsal blade of dermal shoulder girdle (either cleithrum or anterolateral plate)**: 0. spatulate; 1. pointed.

Postcranium (Axial skeleton)

258. **Vertebral column**: 0. unconstricted notochord; 1. disc centra.
    Adelargo schultzei from '?' to '0'
259. **Neural arches and spines**: 0. separate; 1. fused.
    Adelargo schultzei from '?' to '0'

Postcranium (Scale and histology)

260. **Scales**: 0. rhombic; 1. round.
    Adelargo schultzei from '?' to '1'
    Chirodipterus onawayensis from '?' to '1'
    Holodipterus elderae from '?' to '1'
261. **Cosmine on scales**: 0. present; 1. absent.
    Psarolepis from '?' to '0'
    Adelargo schultzei from '?' to '1'
    Chirodipterus onawayensis from '?' to '0'
    Holodipterus elderae from '?' to '1'
    Tarachomylax oepiki from '1' to '0'
262. **Enamel**: 0. single-layered; 1. multi-layered.
263. **Anterodorsal process on scale**: 0. absent; 1. present.
264. **Plicidentine**: 0. absent; 1. present.
265. **Enamel and pore canals**: 0. enamel absent from inner surface of pores; 1. enamel lines portions of pore canal.

Oral elements (Hyoid and Gill skeletons)

266. **Ceratohyal (revised)**: 0. short and stout, 1. long; 2. dumbbell-shaped. We add state 2 (dumbbell-shaped) to apply to some dumbbell-shaped ceratohyal in some lungfishes.
    Griphognathus whitei from '1' to '0'
    Jarvikia arctica from '?' to '1'
    Robinsondipterus longi from '?' to '0'
    Soederberghia groenlandica from '?' to '0'
    Rhinodipterus kimberleyensis from '?' to '0'
    Laccognathus from '0' to '1'
    Holoptychius from '0' to '1'
    Gogonasus from '0' to '1'
267. **Basihyal**: 0. absent, 1. present. **New character**.
268. **Basihyal**: 0. short without denticles; 1. long and denticulated; 2. short and denticulated.
    Youngolepis from '?' to '0'
269. **Articulation facet of the hyomandibular**: 0. single-headed; 1. double-headed.
270. **Oriention of the hyomandibular**: 0. lean anteriorly, 1. relatively vertically, 2. relatively posteriorly, 3. relatively horizontally. **New character**.
271. **Shape of the hyomandibular**: 0. rod-like; 1. triangular. **New character**.
272. **Urohyal morphology**: 0. dorsoventrally compressed and rod-like, may bifurcate posteriorly; 1. vertical plate.
273. **Number of basibranchials**: 0. one; 1. two. **New character**.
274. **Hypobranchials connected with the basibranchials**: 0. three; 1. four; 2. five. **New character**.

275.**Hypobranchial 4**: 0. connected with hypobranchial 3; 1. not connected with hypobranchial 3. **New character**.
276.**Hypohyal connection with basibranchial**: 0. via anterior facet on the basibranchial; 1. via lateral facet on the basibranchial. **New character**.
277.**Hypohyal**: 0. flat; 1. curved and tapering. **New character**.

Taxon list

Adololopas moyasmithae[14]
Adelargo schultzei[15]
Amadeodipterus kencampbelli[16]
Andreyevichthys epitomus[17-19]
Archaeonectes pertusus[20]
Asthenorhynchus meemannae[21,22]
Barwickia downunda[23]
Chirodipterus australis[24-26]
Chirodipterus onawayensis[27]
Chirodipterus wildungensis[28]
Chirodipterus liangchengi[29]
Dipnorhynchus cathlesae[30]
Dipnorhynchus sussmilchi[31-33]
Dipnorhynchus kiandrensis[34]
Dipnorhynchus kurikae[35,36]
Eoctenodus microsoma[37]
Fleurantia denticulata[38]
Gogodipterus paddyensis[39]
Griphognathus minutidens[40,41]
Griphognathus sculpta[40]
Griphognathus whitei[25,42,43]
Grossipterus crassus[44]
Holodipterus elderae[21]
Holodipterus gogoensis[25]
Howidipterus donnae[23]
Ichnomylax kurnai[45]
Iowadipterus halli[46]
Jarvikia arctica[47]
Jessenia concentrica[48]
Melanognathus canadensis[49,50]
Nielsenia nordica[47]
Oervigia nordica[47]
Orlovichthys limnatis[51]
Palaeodaphus insignis[52]
Pentlandia macroptera[53,54]
Phaneropleuron andersoni[55,56]
Pillararhynchus longi[57,58]
Robinsondipterus longi[22]
Scaumenacia curta[59,60]
Soederberghia groenlandica[12,47,61]
Sorbitorhynchus deleaskitus[62,63]
Speonesydrion iani[64,65]

Stomiahykus thlaodus[66]
Tarachomylax oepiki[67]
Uranolophus wyomingensis[68]
Conchopoma gadiforme[69-71]
Delatitia breviceps[72]
Uronemus splendens[71]
Gnathorhiza serrata[73]
Megapleuron zangerli[74]
Palaeophichthys parvulus[75,76]
Parasagenodus sibiricus[77]
Sagenodus inaequalis[78,79]
Straitonia waterstoni[80,81]
Tranodis castrensis[80]
Ctenodus[74,82,83]
Xylognathus macrustenus[84]
Limanichthys fraseri[7]
Apatorhynchus opistheretmus[85]
Erikia jarviki[86]
Dipnotuberculus gnathodus[87]
Cathlorhynchus trismodipterus[88]
Harajicadipterus youngi[89]
Xeradipterus hatcheri[90]
Rhinodipterus ulrichi[91]
Rhinodipterus kimberleyensis[92,93]
Rhinodipterus secans[41]
Westollrhynchus lehmanni[56]
Rhynchodipterus elginensis[94]
Persephonichthys chthonica[95]
Celsiodon ahlbergi[96]
Sinodipterus beibei[97]
Durialepis edentatus[98]
Laccognathus grossi[99,100]
Holoptychius[101,102]
Nasogaluakus chorni[103]
Quebecius quebecensis[101,104]
Glyptolepis groenlandica[3]
Guiyu oneiros[13,105,106]
Porolepis[3,107]
Psarolepis romeri[108-110]
Meemannia eos[111-113]
Youngolepis praecursor[2,114-116]
Powichthys thorsteinssoni[4,117]
Diabolepis speratus[11,118,119]
Dipterus valenciennesi[120-122]
Cheirolepis[123-125]
Raynerius splendens[10]

Supplementary References

1 Chang, M.-M. *The braincase of Youngolepis, a Lower Devonian crossopterygian from Yunnan, south-western China*, University of Stockholm, Department of Geology, (1982).

2 Chang, M.-M. in *Early Vertebrates and Related Problems of Evolutionary Biology* (eds M.-M. Chang, Y.-H. Liu, & G.-R. Zhang) 355 – 378 (Science Press, 1991).

3 Jarvik, E. Middle and Upper Devonian Porolepiformes from East Greenland with special reference to *Glyptolepis groenlandica* n. sp. and a discussion on the structure of the head in the Porolepiformes. *Meddelelser om Grønland* **187**, 1 – 307 (1972).

4 Jessen, H. L. Lower Devonian Porolepiformes from the Canadian Arctic with special reference to *Powichthys thorsteinssoni* Jessen. *Palaeontographica Abteilung A* **167**, 180 – 214 (1980).

5 Zhu, M. & Yu, X.-B. in *Recent Advances in the Origin and Early Radiation of Vertebrates* (eds G. Arratia, M. V. H. Wilson, & R. Cloutier) 271 – 286 (Verlag Dr. Friedrich Pfeil, 2004).

6 Clément, G. & Janvier, P. *Powichthys spitsbergensis* sp. nov., a new member of the Dipnomorpha (Sarcopterygii, lobe-finned fishes) from the Lower Devonian of Spitsbergen, with remarks on basal dipnomorph anatomy. *Fossils & Strata* **50**, 92 – 112 (2004).

7 Challands, T. J. *et al.* A lungfish survivor of the end-Devonian extinction and an Early Carboniferous dipnoan radiation. *Journal of Systematic Palaeontology* **17**, 1825 – 1846 (2019).

8 Schultze, H.-P. A porolepiform rhipidistian from the Lower Devonian of the Canadian Arctic. *Mittelungen aus dem Museum für Naturkunde, Berlin, Geowwissen Reihe* **3**, 99 – 109 (2000).

9 Lu, J., Giles, S., Friedman, M. & Zhu, M. A new stem sarcopterygian illuminates patterns of character evolution in early bony fishes. *Nature Communications* **8**, 1932 (2017).

10 Giles, S., Darras, L., Clement, G., Blieck, A. & Friedman, M. An exceptionally preserved Late Devonian actinopterygian provides a new model for primitive cranial anatomy in ray-finned fishes. *Proceedings of the Royal Society B: Biological Sciences* **282**, 20151485 (2015).

11 Chang, M.-M. & Yu, X.-B. Structure and phylogenetic significance of *Diabolichthys speratus* gen. et sp. nov., a new dipnoan-like form from the Lower Devonian of eastern Yunnan, China. *Proceedings of the Linnean Society of New South Wales* **107**, 171 – 184 (1984).

12 Friedman, M. The interrelationships of Devonian lungfishes (Sarcopterygii: Dipnoi) as inferred from neurocranial evidence and new data from the genus *Soederberghia* Lehman, 1959. *Zoological Journal of the Linnean Society* **151**, 115 – 171 (2007).

13 Qiao, T. & Zhu, M. Cranial morphology of the Silurian sarcopterygian *Guiyu oneiros* (Gnathostomata: Osteichthyes). *Science China Earth Sciences* **53**, 1836-1848 (2010).

14 Campbell, K. S. W. & Barwick, R. E. A new tooth-plated dipnoan from the Upper Devonian Gogo Formation and its relationships. *Memoirs of the Queensland Museum* **42**, 403 – 437 (1998).

15 Johanson, Z. & Ritchie, A. A new late Famennian lungfish from New South Wales, Australia, and its bearing on Australian-Asian terrane relations. *Alcheringa* **24**, 99 – 118 (2000).

16 Young, G. C. & Schultze, H.-P. New osteichthyans (bony fishes) from the Devonian of Central Australia. *Mitt. Mus. Nat.kd. Berl., Geowiss. Reihe* **8**, 13 – 35 (2005).

17 Krupina, N. I. The shoulder girdle and opercular series of *Andreyevichthys epitomus*, a late Devonian Dipnoan from the Tula region of Russia. *Paleontological Journal* **31**, 81 – 86 (1997).

18 Smith, M. M. & Krupina, N. I. Conserved developmental processes constrain evolution of lungfish dentitions. *Journal of Anatomy* **199**, 161 – 168 (2001).

19 Krupina, N. I. A new dipnoan from the Upper Devonian of Tula region. *Paleontological Journal* **3**, 40 – 47 (1987).

20 Meyer, H. v. *Archaeonectes pertusus* aus dem Ober-Devon der Eifel. *Palaeontographica (1846-1933)*, 12 – 13 (1859).

21 Pridmore, P. A., Campbell, K. S. W. & Barwick, R. E. Morphology and phylogenetic position of the holodipteran dipnoans of the Upper Devonian Gogo Formation of northwestern Australia. *Philosophical Transactions of the Royal Society of London, Series B* **334**, 105 ‑ 164 (1994).
22 Long, J. A. in *Morphology, Phylogeny and Paleobiogeography of Fossil Fishes* (eds D. K. Elliott, J. G. Maisey, X. B. Yu, & D. S. Miao) 275-298 (Verlag Dr. Friedrich Pfeil, 2010).
23 Long, J. A. Cranial anatomy of two new Late Devonian lungfishes (Pisces: Dipnoi) from Mount Howitt, Victoria. *Records of the Australian Museum* **44**, 299 ‑ 318 (1992).
24 Henderson, S. A. C. & Challands, T. J. The cranial endocast of the Upper Devonian dipnoan '*Chirodipterus*' *australis*. *PeerJ* **6**, e5148 (2018).
25 Miles, R. S. Dipnoan (lungfish) skulls and the relationships of the group: a study based on new species from the Devonian of Australia. *Zoological Journal of the Linnean Society* **61**, 1 ‑ 328 (1977).
26 Pridmore, P. A. & Barwick, R. E. Post-cranial morphologies of the Late Devonian dipnoans *Griphognathus* and *Chirodipterus* and locomotor implications. *Memoirs of the Association of Australasian Palaeontologists* **15**, 161 ‑ 182 (1993).
27 Schultze, H.-P. A dipterid dipnoan from the Middle Devonian of Michigan, U.S.A. *Journal of Vertebrate Paleontology* **2**, 155 ‑ 162 (1982).
28 Säve-Söderbergh, G. On the skull of *Chirodipterus wildungensis* Gross, an Upper Devonian dipnoan from Wildungen. *Kungliga Svenska Vetenskapsakademiens Handlingar* **4**, 5 ‑ 28 (1952).
29 Song, C. Q. & Chang, M. M. in *Early Vertebrates and Related Problems of Evolutionary Biology* (eds M.M. Chang, Y.H. Liu, & G.R. Zhang) 465 ‑ 476 (Science Press, 1991).
30 Campbell, K. S. W. & Barwick, R. E. A new species of the Devonian lungfish *Dipnorhynchus* from Wee Jasper, New South Wales. *Records of the Australian Museum* **51**, 123 ‑ 140 (1999).
31 Thomson, K. S. & Campbell, K. S. W. The structure and relationships of the primitive Devonian lungfish - *Dipnorhynchus sussmilchi* (Etheridge). *Bulletin of the Peabody Museum of Natural History* **38**, 1 ‑ 109 (1971).
32 Campbell, K. S. W. & Barwick, R. E. The neurocranium of the primitive dipnoan *Dipnorhynchus sussmilchi* (Etheridge). *Journal of Vertebrate Paleontology* **2**, 286 ‑ 327 (1982).
33 Clement, A. M., Challands, T. J., Long, J. A. & Ahlberg, P. E. The cranial endocast of *Dipnorhynchus sussmilchi* (Sarcopterygii: Dipnoi) and the interrelationships of stem-group lungfishes. *PeerJ* **4**, e2539 (2016).
34 Campbell, K. S. W. & Barwick, R. E. A new species of the lungfish *Dipnorhynchus* from New South Wales. *Palaeontology* **25**, 509 ‑ 527 (1982).
35 Campbell, K. S. W. & Barwick, R. E. The braincase, mandible and dental structures of the Early Devonian lungfish *Dipnorhynchus kurikae* from Wee Jasper, New South Wales. *Records of the Australian Museum* **52**, 103 ‑ 128 (2000).
36 Campbell, K. S. W. & Barwick, R. E. An advanced dipnorhynchid lungfish from the Early Devonian of New South Wales, Australia. *Records of the Australian Museum* **37**, 301 ‑ 316 (1985).
37 Long, J. A. A redescription of the lungfish *Eoctenodus* Hills 1929, with reassessment of other Australian records of the genus *Dipterus* Sedgwick & Murchison 1828. *Records of the Western Australian Museum* **13**, 297 ‑ 314 (1987).
38 Graham-Smith, W. & Westoll, T. S. On a new long-headed dipnoan fish from the Upper Devonian of Scaumenac Bay, P.Q., Canada. *Transactions of the Royal Society of Edinburgh: Earth Sciences* **59**, 241 ‑ 268 (1937).
39 Long, J. A. Gogodipterus paddyensis (Miles), gen. nov., a new chirodipterid lungfish from the late Devonian Gogo formation, Western Australia. *Beagle: Records of the Museums and Art Galleries of the Northern Territory* **9**, 11 ‑ 20 (1992).
40 Schultze, H.-P. *Griphognathus* Gross, ein langschnauziger Dipnoer aus dem Oberdevon von

Bergisch-Gladbach (Rheinisches Schiefergebirge) und von Lettland. *Geologica et Palaeontologica* **3**, 21 – 79 (1969).
41 Gross, W. Über Crossopterygier und Dipnoer aus dem baltischen Oberdevon im Zusammenhang einer vergleichenden Untersuchung des Porenkanalsystems paläozoischer Agnathen und Fische. *Kungliga Svenska Vetenskapsakademiens Handlingar* **(4)5**, 1 – 140 (1956).
42 Campbell, K. S. W. & Barwick, R. E. The axial postcranial structure of *Griphognathus whitei* from the Upper Devonian Gogo Formation of Western Australia: comparisons with other Devonian dipnoans. *Records of the Western Australian Museum* **21**, 167 – 201 (2002).
43 Campbell, K. S. W., Barwick, R. E. & Senden, T. J. Development of the posterior endocranium of the Devonian dipnoan *Griphognathus whitei*. *Journal of Vertebrate Paleontology* **32**, 781 – 798 (2012).
44 Gross, W. Die Fische des Baltischen Devons. *Palaeontographica Abteilung A* **79**, 1 – 74 (1933).
45 Long, J. A., Campbell, K. S. W. & Barwick, R. E. A new dipnoan genus, *Ichnomylax*, from the Lower Devonian of Victoria, Australia. *Journal of Vertebrate Paleontology* **14**, 127 – 131 (1994).
46 Schultze, H.-P. A new long-headed dipnoan (Osteichthyes) from the Middle Devonian of Iowa, U.S.A. *Journal of Vertebrate Paleontology* **12**, 42 – 58 (1992).
47 Lehman, J. P. Les dipneustes du Dévonien supérieur du Groenland. *Meddelelser om Grønland* **160**, 1 – 58 (1959).
48 Otto, M. & Bardenheuer, P. Lungfish with dipterid tooth-plates in the Lower Devonian of Central Europe. *Modern Geology* **20**, 341 – 350 (1996).
49 Jarvik, E. On the structure of the lower jaw in dipnoans: with a description of an early Devonian dipnoan from Canada, *Melanognathus canadensis* gen. et sp. nov. *Zoological Journal of the Linnean Society* **47**, 155 – 183 (1967).
50 Schultze, H.-P. *Melanognathus*, a primitive dipnoan from the Lower Devonian of the Canadian Arctic and the interrelationships of Devonian dipnoans. *Journal of Vertebrate Paleontology* **21**, 781 – 794 (2001).
51 Krupina, N. I., Reisz, R. R. & Scott, D. The skull and tooth system of *Orlovichthys limnatis,* a Late Devonian dipnoan from Russia. *Canadian Journal of Earth Sciences* **38**, 1301 – 1311 (2001).
52 Van Beneden, P.-J. & De Koninck, L. Notice sur le *Palaedaphus insignis*. *Bulletin de l'Académie Royal des Lettres et Beaux-Art de Belgique* **17**, 143 – 151 (1864).
53 Jude, E., Johanson, Z., Kearsley, A. & Friedman, M. Early evolution of the lungfish pectoral-fin endoskeleton: evidence from the Middle Devonian (Givetian) Pentlandia macroptera. *Frontiers in Earth Science* **2** (2014).
54 Challands, T. & den Blaauwen, J. A redescription of the Middle Devonian dipnoan *Pentlandia macroptera* Traquair, 1889, and an assessment of the Phaneropleuridae. *Zoological Journal of the Linnean Society* **180**, 414 – 460 (2017).
55 Huxley, T. H. Preliminary essay upon the systematic arrangement of the fishes of the Devonian epoch. *Mem. Geol. Surv. United Kingdom, decade 10*, 1 – 40 (1861).
56 Westoll, T. S. in *Genetics, Paleontology and Evolution* (eds G. L. Jepsen, E. Mayr, & G. G. Simpson) 121 – 184 (Princeton University Press, 1949).
57 Campbell, K. S. W. & Barwick, R. E. Paleozoic Dipnoan Phylogeny: Functional Complexes and Evolution Without Parsimony. *Paleobiology* **16**, 143 – 169 (1990).
58 Barwick, R. E. & Campbell, K. S. W. A Late Devonian dipnoan, *Pillararhynchus*, from Gogo, Western Australia, and its relationships. *Palaeontographica Abteilung A* **239**, 1 – 42 (1996).
59 Cloutier, R. Morphologie et variations du toit crânien du dipneuste *Scaumenacia curta* (Whiteaves) (Sarcopterygii), du Dévonien supérieur du Québec. *Geodiversitas* **19**, 61 – 105 (1997).
60 Béchard, I. *Développement larvaire et juvénile de Scaumenacia curta (Sarcopterygii: Dipnoi)*

du Dévenien supérieur de Miguasha, Québec, Canada, Université du Québec à Rimouski (2011).
61 Friedman, M. Cranial structure in the Devonian lungfish *Soederberghia groenlandica* and its implications for the interrelationships of ‘rhynchodipterids’. *Earth and Environmental Science Transactions of the Royal Society of Edinburgh* **98**, 179–198 (2008).
62 Wang, S.-T., Drapala, V., Barwick, R. E. & Campbell, K. S. W. The dipnoan species *Sorbitorhynchus deleaskitus*, from the Lower Devonian of Guangxi, China. *Philosophical Transactions of the Royal Society of London, Series B* **340**, 1–24 (1993).
63 Kemp, A. Possible pathology in the snout and lower jaw of the Chinese Devonian lungfish, *Sorbitorhynchus deleaskitus* (Osteichthyes: Dipnoi). *Journal of Vertebrate Paleontology* **14**, 453–458 (1995).
64 Campbell, K. S. W. & Barwlck, R. E. The structure and stratigraphy of Speonesydrion from New South Wales, Australia, and the dentition of primitive dipnoans. *Palaontologische Zeitschrift* **81/2**, 146–159 (2007).
65 Campbell, K. S. W. & Barwick, R. E. *Speonysedrion*, an Early Devonian dipnoan with primitive tooth plates. *Palaeo Ichthyologica* **2**, 1–48 (1984).
66 Bernacsek, G. M. A lungfish cranium from the Middle Devonian of the Yukon Territory, Canada. *Palaeontographica Abteilung A* **157**, 175–200 (1977).
67 Barwick, R. E., Campbell, K. S. W. & Mark-Kurik, E. *Tarachomylax*: A new Early Devonian dipnoan from Severnaya Zemlya, and its place in the evolution of the Dipnoi. *Geobios* **30**, 45–73 (1997).
68 Denison, R. H. Early Devonian lungfishes from Wyoming, Utah, and Idaho. *Fieldiana Geology* **17(4)**, 353–413 (1968).
69 Heidtke, U. Über Neufunde von *Conchopoma gadiforme* Kner (Dipnoi: Pisces). *Paläontologische Zeitschrift* **60**, 299–312 (1986).
70 Schultze, H.-P. Die Lungenfisch-Gattung *Conchopoma* (Pisces, Dipnoi). *Senckenbergiana Lethaea* **56**, 191–231 (1975).
71 Watson, D. M. S. & Gill, E. L. The structure of certain Palaeozoic Dipnoi. *Zoological Journal of the Linnean Society* **35**, 163 (1923).
72 Long, J. A. & Campbell, K. S. W. A new lungfish from the Lower Carboniferous of Victoria, Australia. *Proceedings of the Royal Society of Victoria* **97**, 87–93 (1985).
73 Carlson, K. J. The skull morphology and estivation burrows of the Permian lungfish, *Gnathoriza serrata*. *Journal of Geology* **76**, 641–663 (1968).
74 Schultze, H.-P. *Megapleuron zangerli* a new dipnoan from the Pennsylvanian, Illinois. *Fieldiana Geology* **33**, 375–396 (1977).
75 Kemp, A. On the generic status of *Palaeophichthys parvulus* Eastman 1908 and *Monongahela stenodonta* Lund 1970 (Osteichthyes: Dipnoi). *Annals of Carnegie Museum* **67**, 225–243 (1998).
76 Schultze, H.-P. *Paleophichthys parvulus* Eastman, 1908, a gnathorhizid dipnoan from the Middle Pennsylvanian of Illinois, USA. *Annals of Carnegie Museum* **63**, 105–113 (1994).
77 Vorobyeva, E. I. New genus of dipnoan from the Emyaksin Formation of Yakutia, *Paleontological Journal* **6**, 229–234 (1972).
78 Smith, M. M. Sturcture and histogenesis of tooth plates in *Sagenodus inaequalis* Owen considered in relation to the phylogeny of post-Devonian dipnoans. *Proceedings of the Royal Society of London Series B-Biological Sciences* **204**, 15–39 (1979).
79 Beeby, E. L., Smithson, T. R. & Clack, J. A. Systematics and description of the lungfish genus *Sagenodus* from the Carboniferous of the UK. *Earth and Environmental Science Transactions of the Royal Society of Edinburgh* **111**, 47–74 (2020).
80 Thomson, K. S. On the relationships of certain Carboniferous Dipnoi; with descriptions of four new forms. *Proceedings of the Royal Society of Edinburgh, B* **69**, 221–245 (1965).
81 Sharp, E. & Clack, J. Redescription of the lungfish *Straitonia waterstoni* from the Viséan of Lothian, Scotland. *Earth and Environmental Science Transactions of the Royal Society of*

Edinburgh **102**, 179–190 (2012).
82 Sharp, E. L. & Clack, J. A. A review of the Carboniferous lungfish genus *Ctenodus* Agassiz, 1838 from the United Kingdom, with new data from an articulated specimen of *Ctenodus interruptus* Barkas, 1869. *Earth and Environmental Science Transactions of The Royal Society of Edinburgh* **104**, 169–204 (2013).
83 Kemp, A. *Ctenodus boudariensis* nov. sp., a ctenodont lungfish from the mid-Viséan Ducabrook Formation of Queensland, Australia. *Alcheringa: An Australasian Journal of Palaeontology* **43**, 423–429 (2019).
84 Smithson, T. R., Richards, K. R., Clack, J. A. & Johanson, Z. Lungfish diversity in Romer's Gap: reaction to the end-Devonian extinction. *Palaeontology* **59**, 29–44 (2016).
85 Friedman, M. & Daeschler, E. B. Late Devonian (Famennian) lungfishes from the Catskill Formation of Pennsylvania, USA. *Palaeontology* **49**, 1167–1183 (2006).
86 Chang, M.-M. & Wang, J.-Q. A new Emsian dipnorhynchid (Dipnoi from Guangnan, southeastern Yunnan, China. *Geobios* **19**, 233–239 (1995).
87 Campbell, K. S. W., Barwick, R. E., Chatterton, B. D. E. & Smithson, T. R. A new Middle Devonian dipnoan from Morocco: structure and histology of the dental plates. *Records of the Western Australian Museum* **21**, 39–61 (2002).
88 Campbell, K. S. W., Barwick, R. E. & Senden, T. J. Evolution of dipnoans (lungfish) in the Early Devonian of southeastern Australia. *Alcheringa* **33**, 59–78 (2009).
89 Clement, A. M. A new genus of lungfish from the Givetian (Middle Devonian) of Central Australia. *Acta Palaeontologica Polonica* **54**, 615–626 (2009).
90 Clement, A. M. & Long, J. A. *Xeradipterus hatcheri,* a new dipnoan from the Late Devonian (Frasnian) Gogo Formation, Western Australia, and other new holodontid material. *Journal of Vertebrate Paleontology* **30**, 681–695 (2010).
91 Ørvig, T. New finds of Acanthodians, Arthrodires, Crossoptergians, Ganoids and Dipnoans in the upper Middle Devonian Calcareous Flags (Oberer Plattenkalk) of the Bergisch Gladbach-Paffrath Trough (Part 2). *Paläontologische Zeitschrift* **35**, 10–27 (1961).
92 Clement, A. M. A new species of long-snouted lungfish from the Late Devonian of Australia, and its functional and biogeographical implications. *Palaeontology* **55**, 51–71 (2012).
93 Clement, A. M. & Ahlberg, P. E. The first virtual cranial endocast of a lungfish (Sarcopterygii: Dipnoi). *PLoS One* **9**, e113898 (2014).
94 Säve-Söderbergh, G. On *Rhynchodipterus elginensis* n. g., n. sp., representing a new group of dipnoan-lilke Choanata from the Upper Devonian of East Greenland and Scotland. A preliminary note. *Arkiv för Zoologi* **29B**, 1–8 (1937).
95 Pardo, J. D., Huttenlocker, A. K. & Small, B. J. An exceptionally preserved transitional lungfish from the Lower Permian of Nebraska, USA, and the origin of modern lungfishes. *PLoS ONE* **9**, e108542 (2014).
96 Clack, J. A., Challands, T. J., Smithson, T. R., Smithson, K. Z. & Ruta, M. Newly recognized Famennian lungfishes from East Greenland reveal tooth plate diversity and blur the Devonian–Carboniferous boundary. *Papers in Palaeontology* **5**, 261–279 (2019).
97 Qiao, T. & Zhu, M. A new tooth-plated lungfish from the Middle Devonian of Yunnan, China, and its phylogenetic relationships. *Acta Zoologica* **90**, 236–252 (2009).
98 Mondéjar‐Fernández, J., Friedman, M., Giles, S. & Cavin, L. Redescription of the cranial skeleton of the Early Devonian (Emsian) sarcopterygian *Durialepis edentatus* Otto (Dipnomorpha, Porolepiformes). *Papers in Palaeontology* **7**, 789–806 (2021).
99 Vorobyeva, E. I. A new species of *Laccognathus* (Porolepiform Crossopterygii) from the Devonian of Latvia. *Paleontological Journal* **40**, 312–322.
100 Downs, J. P., Daeschler, E. B., Jenkins, F. A. & Shubin, N. H. A new species of *Laccognathus* (Sarcopterygii, Porolepiformes) from the Late Devonian of Ellesmere Island, Nunavut, Canada. *Journal of Vertebrate Paleontology* **31**, 981–996 (2011).

101 Cloutier, R. & Schultze, H.-P. in *Devonian Fishes and Plants of Miguasha, Quebec, Canada* (eds H-P Schultze & R Cloutier) 248–270 (Verlag Dr. F. Pfeil, 1996).
102 Downs, J. P., Daeschler, E. B., Jenkins, F. A. & Shubin, N. H. *Holoptychius bergmanni* sp. nov. (Sarcopterygii, Porolepiformes) from the Upper Devonian of Nunavut, Canada, and a review of *HoloptychiusTaxonomy*. *Proceedings of the Academy of Natural Sciences of Philadelphia* **162**, 47–59 (2013).
103 Schultze, H.-P. A porolepiform rhipidistian from the Lower Devonian of the Canadian Arctic. *Mitt. Mus. Nat.kd. Berl., Geowiss. Reihe* **3**, 99–109 (2000).
104 Schultze, H.-P. & Arsenault, M. *Quebecius quebecensis* (Whiteaves), a porolepiform crossopterygian (Pisces) from the Late Devonian of Quebec, Canada. *Canadian Journal of Earth Sciences* **24**, 2351–2361 (1987).
105 Zhu, M. *et al.* The oldest articulated osteichthyan reveals mosaic gnathostome characters. *Nature* **458**, 469–474 (2009).
106 Cui, X., Qiao, T. & Zhu, M. Scale morphology and squamation pattern of *Guiyu oneiros* provide new insights into early osteichthyan body plan. *Scientific Reports* **9**, 4411, (2019).
107 Kulczycki, J. *Porolepis* (Crossopterygii) from the Lower Devonian of the Holy Cross Mountains. *Acta Palaeontologica Polonica* **5**, 65–106 (1960).
108 Yu, X.-B. A new porolepiform-like fish, *Psarolepis romeri*, gen. et sp. nov. (Sarcopterygii, Osteichthyes) from the Lower Devonian of Yunnan, China. *Journal of Vertebrate Paleontology* **18**, 261–274 (1998).
109 Qu, Q. M., Zhu, M. & Wang, W. Scales and dermal skeletal histology of an early bony fish *Psarolepis romeri* and their bearing on the evolution of rhombic scales and hard tissues. *PLoS One* **8**, e61485 (2013).
110 Zhu, M., Yu, X.-B. & Janvier, P. A primitive fossil fish sheds light on the origin of bony fishes. *Nature* **397**, 607–610 (1999).
111 Zhu, M., Yu, X.-B., Wang, W., Zhao, W.-J. & Jia, L.-T. A primitive fish provides key characters bearing on deep osteichthyan phylogeny. *Nature* **441**, 77–80 (2006).
112 Zhu, M., Wang, W. & Yu, X.-B. in *Morphology, Phylogeny and Paleobiogeography of Fossil Fishes* (eds D. K. Elliott, J. G. Maisey, X.-B. Yu, & D.-S. Miao) 199–214 (Verlag Dr. Friedrich Pfeil, 2010).
113 Lu, J., Giles, S., Friedman, M., den Blaauwen, J. L. & Zhu, M. The oldest actinopterygian highlights the cryptic early history of the hyperdiverse ray-finned fishes. *Current Biology* **26**, 1602–1608 (2016).
114 Chang, M.-M. & Yu, X.-B. A new crossopterygian, *Youngolepis praecursor*, gen. et sp. nov., from Lower Devonian of eastern Yunnan, China. *Scientia Sinica* **24**, 89–99 (1981).
115 Chang, M.-M. *The braincase of Youngolepis, a Lower Devonian crossopterygian from Yunnan, south-western China*. (University of Stockholm, Department of Geology, 1982).
116 Zhu, M. & Fan, J. H. *Youngolepis* from the Xishancun Formation (Early Lochkovian) of Qujing, China. *Geobios* **19**, 293–299 (1995).
117 Jessen, H. L. in *Problèmes actuels de Paléontologie-Evolution des Vertébrés* Vol. 218 (ed J P Lehman) 213–222 (Colloques Internationaux du Centre National de la Recherche Scientifique, 1975).
118 Smith, M. M. & Chang, M.-M. The dentition of *Diabolepis speratus* Chang and Yu, with further consideration of its relationships and the primitive dipnoan dentition. *Journal of Vertebrate Paleontology* **10**, 420–433 (1990).
119 Chang, M.-M. *Diabolepis* and its bearing on the relationships between porolepiforms and dipnoans. *Bulletin du Muséum national d'Histoire naturelle, Paris 4e sér., Section C* **17**, 235–268 (1995).
120 Ahlberg, P. E. & Trewin, N. H. The postcranial skeleton of the Middle Devonian lungfish *Dipterus valenciennesi*. *Transactions of the Royal Society of Edinburgh: Earth Sciences* **85**, 159–

175 (1995).
121 Campbell, K. S. W., Barwick, R. E. & den Blaauwen, J. L. Structure and function of the shoulder girdle in dipnoans: new material from *Dipterus valenciennesi*. *Senckenbergiana lethaea* **86**, 77–91 (2006).
122 Challands, T. J. & Johanson, Z. The cranial endocast of the Middle Devonian dipnoan *Dipterus valenciennesi* and a fossilized dipnoan otoconial mass. *Papers in Palaeontology* **1**, 289–317 (2015).
123 Pearson, D. M. & Westoll, T. S. The Devonian actinopterygian *Cheirolepis* Agassiz. *Transactions of the Royal Society of Edinburgh: Earth Sciences* **70**, 337–399 (1979).
124 Arratia, G. & Cloutier, R. in *Devonian Fishes and Plants of Miguasha, Quebec, Canada* (eds H.-P. Schultze & R. Cloutier) 165–197 (Verlag Dr Friedrich Pfeil, 1996).
125 Giles, S. *et al.* Endoskeletal structure in *Cheirolepis* (Osteichthyes, Actinopterygii), an early ray-finned fish. *Palaeontology* **58**, 849–870 (2015).
