# Supplementary material for: The rapid evolution of lungfish durophagy
Source: Nat Commun. 2022 May 2;13:2390. doi: 10.1038/s41467-022-30091-3 (PMC9061808; doi:10.1038/s41467-022-30091-3)
Supplement: Supplementary file 3 — Description of Additional Supplementary Files [file 41467_2022_30091_MOESM3_ESM.pdf]

Descriptions of Additional Supplementary Files

Supplementary Data 1: Contains the morphological matrix (in .nex and .tnt formats) and a .tre file of the parsimony strict consensus tree analyzed in TNT. The morphological matrix file including 88 extant taxa and 277 morphological characters. The .nex file can be opened by Mesquite, the .tnt file can be opened by TNT, and the .tre file can be opened by Figtree.

Supplementary Software: Contains codes for Maximum-likelihood analysis (Maximum-likelihood_analysis folder) and MrBayes tip-dating analyses (MrBayes tip dating analyses folder). The Maximum-likelihood_analysis folder has a .phy file (matrix) can opened by IQtree and a .txt file (code). The MrBayes tip dating analyses folder contains 12 subfolders, each corresponding to 12 MrBayes tip-dating analyses: we used independent gamma rate (IGR), independent lognormal (ILN) and autocorrelated-rates (TK02) models, each with four separate analyses (unpartitioned matrix with no topological constraint topology, 2-partition matrix with no topological constraint topology, unpartitioned matrix with topological constraints reflecting the strict consensus tree from our parsimony analysis, 2-partition matrix with topological constraints reflecting the strict consensus tree from our parsimony analysis). Each of the 12 subfolders contains codes and results of the corresponding analysis. In addition, Supplementary Software folder has a R script that utilized the best-scoring maximum-likelihood morphological tree as input, helping to derive the clock rate prior.
