# Supplementary figures and images for: The rapid evolution of lungfish durophagy
Source: Nat Commun. 2022 May 2;13:2390. doi: 10.1038/s41467-022-30091-3 (PMC9061808; doi:10.1038/s41467-022-30091-3)

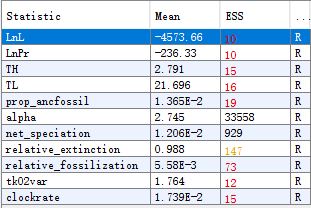

Supplement: Supplementary file 5 — Supplementary Software [file 41467_2022_30091_MOESM5_ESM.zip › Supplementary Software/MrBayes_tip_dating_analyses/unpartition_constraint_TK02_(not_converged∩╝ë/ess.JPG]

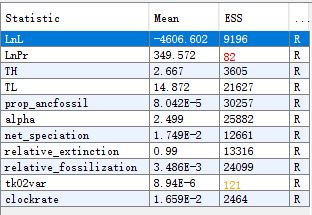

Supplement: Supplementary file 5 — Supplementary Software [file 41467_2022_30091_MOESM5_ESM.zip › Supplementary Software/MrBayes_tip_dating_analyses/unpartition_without_constraint_TK02_(not_converged∩╝ë/ess.JPG]

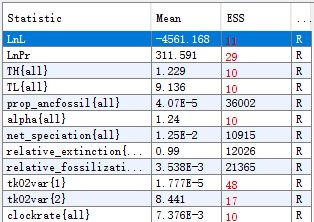

Supplement: Supplementary file 5 — Supplementary Software [file 41467_2022_30091_MOESM5_ESM.zip › Supplementary Software/MrBayes_tip_dating_analyses/2_partitions_constraint_TK02_(not_converged∩╝ë/ess.JPG]

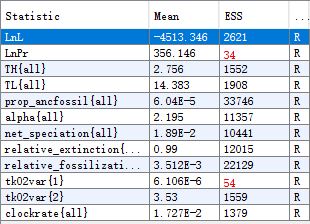

Supplement: Supplementary file 5 — Supplementary Software [file 41467_2022_30091_MOESM5_ESM.zip › Supplementary Software/MrBayes_tip_dating_analyses/2_partitions_without_constraint_TK02_(not_converged)/ess.JPG]
