# Supplementary material for: The rapid evolution of lungfish durophagy
Source: Nat Commun. 2022 May 2;13:2390. doi: 10.1038/s41467-022-30091-3 (PMC9061808; doi:10.1038/s41467-022-30091-3)
Supplement: Supplementary file 6 — Reporting Summary [file 41467_2022_30091_MOESM6_ESM.pdf]

nature portfolio

Corresponding author(s): Min Zhu

Last updated by author(s): Mar 17, 2022

Reporting Summary

Nature Portfolio wishes to improve the reproducibility of the work that we publish. This form provides structure for consistency and transparency in reporting. For further information on Nature Portfolio policies, see our Editorial Policies and the Editorial Policy Checklist.

Statistics

For all statistical analyses, confirm that the following items are present in the figure legend, table legend, main text, or Methods section.

n/a	Confirmed	
[x]	[ ]	The exact sample size (n) for each experimental group/condition, given as a discrete number and unit of measurement
[x]	[ ]	A statement on whether measurements were taken from distinct samples or whether the same sample was measured repeatedly
[x]	[ ]	The statistical test(s) used AND whether they are one- or two-sided<br>*Only common tests should be described solely by name; describe more complex techniques in the Methods section.*
[x]	[ ]	A description of all covariates tested
[x]	[ ]	A description of any assumptions or corrections, such as tests of normality and adjustment for multiple comparisons
[x]	[ ]	A full description of the statistical parameters including central tendency (e.g. means) or other basic estimates (e.g. regression coefficient) AND variation (e.g. standard deviation) or associated estimates of uncertainty (e.g. confidence intervals)
[x]	[ ]	For null hypothesis testing, the test statistic (e.g. F, t, r) with confidence intervals, effect sizes, degrees of freedom and P value noted<br>*Give P values as exact values whenever suitable.*
[ ]	[x]	For Bayesian analysis, information on the choice of priors and Markov chain Monte Carlo settings
[x]	[ ]	For hierarchical and complex designs, identification of the appropriate level for tests and full reporting of outcomes
[x]	[ ]	Estimates of effect sizes (e.g. Cohen's d, Pearson's r), indicating how they were calculated

Our web collection on statistics for biologists contains articles on many of the points above.

Software and code

Policy information about availability of computer code

Data collection	The specimens were scanned using a Nikon XT H 225ST industrial µCT scanner at the CTEES facility, Department of Earth and Environmental Sciences, University of Michigan. Tomographic data were segmented using the software Mimics 22.0, with images of models rendered in Blender 2.90.1. The phylogenetic matrix used in the analyses were compiled in Mesquite 3.6 (Open Source). We performed Bayesian tip-dating analyses in Mrbayes 3.2.8. Convergence of parameters was identified using Tracer 1.7.
Data analysis	A parsimony analysis was conducted in TNT 1.5. We performed a maximum-likelihood non-clock analysis in IQ-TREE 1.6.12. We performed Bayesian tip-dating analyses in Mrbayes 3.2.8. The time-dependent morphological rates were reported in R 4.0.1.

For manuscripts utilizing custom algorithms or software that are central to the research but not yet described in published literature, software must be made available to editors and reviewers. We strongly encourage code deposition in a community repository (e.g. GitHub). See the Nature Portfolio guidelines for submitting code & software for further information.

Data

Policy information about availability of data

All manuscripts must include a data availability statement. This statement should provide the following information, where applicable:

- Accession codes, unique identifiers, or web links for publicly available datasets
- A description of any restrictions on data availability
- For clinical datasets or third party data, please ensure that the statement adheres to our policy

The CT data and 3D models generated in this study have been deposited in the figshare database with the link: https://figshare.com/s/a33db8ae0d64133b92d3. The phylogenetic data generated in this study are provided in the Supplementary Data and Software files. The additional notes generated in this study are provided in the Supplementary Information file.

Field-specific reporting

Please select the one below that is the best fit for your research. If you are not sure, read the appropriate sections before making your selection.

☐ Life sciences ☐ Behavioural & social sciences ☒ Ecological, evolutionary & environmental sciences

For a reference copy of the document with all sections, see nature.com/documents/nr-reporting-summary-flat.pdf

Ecological, evolutionary & environmental sciences study design

All studies must disclose on these points even when the disclosure is negative.

Study description	This study described two new specimens of Devonian sarcopterygian, Youngolepis, and analyzed the evolution rates of lungfishes.
Research sample	Two articulated specimens of Devonian sarcopterygian, Youngolepis praecursor, are both composed of a nearly complete head and partial body. We chose them because they are exceptionally articulated preserved specimens of Youngolepis that is the most primitive lungfish. This material derives from the argillaceous limestone of the Xitun Formation (Early Devonian: late Lochkovian) in Qujing, Yunnan, China
Sampling strategy	We sorted out thousands of fossils of Youngolepis, and finally chose the two articulated specimens of Youngolepis.
Data collection	The morphological data of the fossil specimens was collected and codified as discrete characters by Xindong Cui. CT data and 3D reconstructions were collected based on CT scanning and digital segmentation generated by Matt Friedman and Xindong Cui.
Timing and spatial scale	The fossil specimens were discovered in 2017 from the outcrops (10 m long and 2 m wide) near the Xitun village (103.7054° E, 25.5214° N, 1874m), Qujing, Yunnan, China. They were prepared by a professional technician from the Institute of Vertebrate Paleontology and Paleoanthropology, under the supervision of the authors from 2017 to early 2019. Since then, the specimens were studied by the authors.
Data exclusions	No data is excluded.
Reproducibility	Replicated experiments are unnecessary in paleontology.
Randomization	No randomization procedure was applicable because it was not necessary for the type of study we conducted.
Blinding	Blinding was not relevant for data collection as samples were selected based on their location within the stratigraphy. Blinding was also not relevant for downstream analysis as previously established analysis pipelines was used for the processing of the data.

Did the study involve field work? ☒ Yes ☐ No

Field work, collection and transport

Field conditions	Annual average temperature is about 14.5°C and annual precipitation is about 590mm.
Location	Xitun Village (103.7054° E, 25.5214° N, 1874m), Qujing, Yunnan, China.
Access & import/export	Access and collection was carried out with permission of Qujing government and the government of China.
Disturbance	No disturbances were caused during the study.

Reporting for specific materials, systems and methods

We require information from authors about some types of materials, experimental systems and methods used in many studies. Here, indicate whether each material, system or method listed is relevant to your study. If you are not sure if a list item applies to your research, read the appropriate section before selecting a response.

Materials & experimental systems

n/a	Involved in the study	
☒	☐	Antibodies
☒	☐	Eukaryotic cell lines
☐	☒	Palaeontology and archaeology
☒	☐	Animals and other organisms
☒	☐	Human research participants
☒	☐	Clinical data
☒	☐	Dual use research of concern

Methods

n/a	Involved in the study	
☒	☐	ChIP-seq
☒	☐	Flow cytometry
☒	☐	MRI-based neuroimaging

Palaeontology and Archaeology

Specimen provenance	Access and collection was carried out with permission of Qujing government and the government of China. Related research about the specimens are permitted by IVPP.
Specimen deposition	The specimens are housed at the Institute of Vertebrate Paleontology and Paleoanthropology, Chinese Academy of Sciences.
Dating methods	We dated that the beds yielded Youngolepis were the Early Devonian (late Lochkovian, ~415 Ma) according to the study of Zhao et al., Sci. China Earth Sci. 64, 1–14 (2021). They used everal geochemical indicators including the values and curves of δ 13Corg, δ13Ccarb and TOC are obtained from the continuous SDB sequence in Dahe, Yiliang County, East Yunnan. They carried out the experiments at the State Key Laboratory of Isotope Geochemistry, Guangzhou Institute of Geochemistry, Chinese Academy of Sciences.

☒ Tick this box to confirm that the raw and calibrated dates are available in the paper or in Supplementary Information.

Ethics oversight	No ethical permits were needed for this study because these specimens are fossil specimens.

Note that full information on the approval of the study protocol must also be provided in the manuscript.
